# Real-world assessment of immunogenicity in immunocompromised individuals following SARS-CoV-2 mRNA vaccination: a one-year follow-up of the prospective clinical trial COVAXID

**DOI:** 10.1016/j.ebiom.2023.104700

**Published:** 2023-07-13

**Authors:** Puran Chen, Peter Bergman, Ola Blennow, Lotta Hansson, Stephan Mielke, Piotr Nowak, Gunnar Söderdahl, Anders Österborg, C.I. Edvard Smith, Jan Vesterbacka, David Wullimann, Angelica Cuapio, Mira Akber, Gordana Bogdanovic, Sandra Muschiol, Mikael Åberg, Karin Loré, Margaret Sällberg Chen, Marcus Buggert, Per Ljungman, Soo Aleman, Hans-Gustaf Ljunggren

**Affiliations:** a Department of Medicine Huddinge, Center for Infectious Medicine, Karolinska Institutet, Stockholm, Sweden; b Department of Infectious Diseases, Karolinska University Hospital, Stockholm, Sweden; c Department of Laboratory Medicine, Clinical Immunology, Karolinska Institutet, Stockholm, Sweden; d Department of Transplantation, Karolinska University Hospital, Stockholm, Sweden; e Department of Clinical Science, Intervention and Technology, Karolinska Institutet, Stockholm, Sweden; f Department of Hematology, Karolinska University Hospital, Stockholm, Sweden; g Department of Oncology-Pathology, Karolinska Institutet, Stockholm, Sweden; h Department of Cellular Therapy and Allogeneic Stem Cell Transplantation (CAST), Karolinska University Hospital Huddinge, Karolinska Comprehensive Cancer Center, Stockholm, Sweden; i Department of Laboratory Medicine, Biomolecular and Cellular Medicine, Karolinska Institutet, Stockholm, Sweden; j Department of Medicine Huddinge, Infectious Diseases, Karolinska Institutet, Stockholm, Sweden; k Department of Clinical Microbiology, Karolinska University Hospital, Stockholm, Sweden; l Department of Microbiology, Tumor and Cell Biology, Karolinska Institutet, Stockholm, Sweden; m Department of Medical Sciences, Clinical Chemistry, Science for Life Laboratory, Uppsala University, Uppsala, Sweden; n Department of Medicine Solna, Karolinska Institutet, Stockholm, Sweden; o Department of Dental Medicine, Karolinska Institutet, Stockholm, Sweden; p Division of Hematology, Department of Medicine Huddinge, Karolinska Institutet, Stockholm, Sweden

**Keywords:** SARS-CoV-2, COVID-19, mRNA vaccine, Clinical study, Primary immunodeficiency disease, HIV, Solid organ transplantation, Haematopoietic stem cell transplantation, Chronic lymphocytic leukemia

## Abstract

**Background:**

Immunocompromised patients have varying responses to SARS-CoV-2 mRNA vaccination. However, there is limited information available from prospective clinical trial cohorts with respect to long-term immunogenicity-related responses in these patient groups following three or four vaccine doses, and in applicable cases infection.

**Methods:**

In a real-world setting, we assessed the long-term immunogenicity-related responses in patients with primary and secondary immunodeficiencies from the prospective open-label clinical trial COVAXID. The original clinical trial protocol included two vaccine doses given on days 0 and 21, with antibody titres measured at six different timepoints over six months. The study cohort has subsequently been followed for one year with antibody responses evaluated in relation to the third and fourth vaccine dose, and in applicable cases SARS-CoV-2 infection. In total 356/539 patients were included in the extended cohort. Blood samples were analysed for binding antibody titres and neutralisation against the Spike protein for all SARS-CoV-2 variants prevailing during the study period, including Omicron subvariants. SARS-CoV-2 infections that did not require hospital care were recorded through quarterly in-person, or phone-, interviews and assessment of IgG antibody titres against SARS-CoV-2 Nucleocapsid. The original clinical trial was registered in EudraCT (2021-000175-37) and clinicaltrials.gov (NCT04780659).

**Findings:**

The third vaccine dose significantly increased Spike IgG titres against all the SARS-CoV-2 variants analysed in all immunocompromised patient groups. Similarly, neutralisation also increased against all variants studied, except for Omicron. Omicron-specific neutralisation, however, increased after a fourth dose as well as after three doses and infection in many of the patient subgroups. Noteworthy, however, while many patient groups mounted strong serological responses after three and four vaccine doses, comparably weak responders were found among patient subgroups with specific primary immunodeficiencies and subgroups with immunosuppressive medication.

**Interpretation:**

The study identifies particularly affected patient groups in terms of development of long-term immunity among a larger group of immunocompromised patients. In particular, the results highlight poor vaccine-elicited neutralising responses towards Omicron subvariants in specific subgroups. The results provide additional knowledge of relevance for future vaccination strategies.

**Funding:**

The present studies were supported by grants from the 10.13039/501100004359Swedish Research Council, the 10.13039/501100004063Knut and Alice Wallenberg Foundation, Nordstjernan AB, Region Stockholm, and 10.13039/501100004047Karolinska Institutet.

**Research in context**

**Evidence before this study**

Immunocompromised patients have increased risk for severe COVID-19 and COVID-19-associated death and respond variably to SARS-CoV-2 mRNA vaccination. Limited information comparing different immunocompromised patient groups with respect to long-term immunogenicity following three and four vaccine doses, particularly in studies that take SARS-CoV-2 infection into consideration, is available. In support of this notion, on March 27, 2023, we searched PubMed for “Clinical Trials” with the following search criteria: (“SARS-CoV-2” OR “COVID-19”) AND (“immunocompromised” OR “immunodeficient”) AND (“vaccination”) AND (“mRNA”). The query returned eight clinical trials. None of the clinical trials identified investigated long-term immunity (>2 months following primary vaccination), the effect of booster doses, and SARS-CoV-2 infection in relation to immunogenicity. Notably, however, other related search criteria returned additional studies related to the present topic.

**Added value of this study**

The present study, involving 356 study subjects from the COVAXID cohort, followed five major immunocompromised patient groups and respective subgroups as well as healthy controls over one year in a real-world setting. The study subjects were followed since the very first vaccine dose with longitudinal blood samplings and documentation of booster mRNA vaccine doses as well as SARS-CoV-2 infections following an initial two-dose regimen of BNT162b2 mRNA vaccination. In terms of long-term antibody responses to SARS-CoV-2 mRNA vaccination, our data grossly identifies three classes of immunocompromised patients broadly defined by their serological response patterns; 1) strong responders, e.g., patients having undergone HSCT and people living with HIV (PLWH), 2) weak responders, e.g., patients having undergone SOT and treated with MMF, patients with CVID, and patients with CLL treated with ibrutinib, and 3) non-responders, e.g., patients with XLA. The “strong responders” showed responses equivalent to healthy controls over time. Taken together, the study identifies particularly vulnerable patient groups in terms of immunogenicity-related responses among a large group of patients with primary and secondary immunodeficiencies.

**Implications of all the available evidence**

We here provide a comprehensive, longitudinal assessment of immunogenicity-related responses in a broad range of immunocompromised patient groups. The study allows temporal as well as comparative assessments across many patient groups in a real-world clinical study setting. The findings show that several immunocompromised patient groups need additional booster vaccine doses compared to healthy controls to reach similar levels of immunogenicity. In parallel, subgroups of non- or weak-responders are identified.

## Introduction

Coronavirus disease 2019 (COVID-19) was declared a pandemic by the World Health Organization (WHO) in March 2020.^1^^,^^2^ The pandemic subsequently evolved with the emergence of several new SARS-CoV-2 variants-of-concern (VOC).^3^^,^^4^ Immunocompromised patient groups were quickly identified as high-risk groups for severe COVID-19 and death.^5^

Various platforms were employed to develop vaccines against SARS-CoV-2, including new mRNA-based platforms which demonstrated good safety profiles and high efficacy preventing severe COVID-19 and associated death.^6^, ^7^, ^8^ However, pivotal vaccine trials did not include immunocompromised patient groups, creating an unmet need for prospective clinical trials that evaluated safety and immunological responses in these patient populations. As a result, the COVAXID clinical trial was initiated to address the safety and immunogenicity of the BNT162b2 mRNA vaccine in patients with primary or secondary immunodeficiencies, including those with human immunodeficiency virus (HIV), allogeneic hematopoietic stem cell transplant (HSCT) recipients, solid organ transplant (SOT) recipients, and patients with chronic lymphocytic leukaemia (CLL).^9^ Early data from the COVAXID clinical trial showed that the BNT162b2 mRNA vaccine was generally safe, though some adverse immune activation phenomena were observed. Furthermore, varying degrees of antibody (Ab) responses were observed two weeks following the second dose in the different immunocompromised patient groups.^9^

While short- and longer-term immunogenicity data from other clinical studies assessing the immunogenicity of SARS-CoV-2 vaccination in immunocompromised patient groups have been reported,^10^, ^11^, ^12^, ^13^, ^14^, ^15^, ^16^, ^17^ studies of long-term immunogenicity-related responses among larger sets of immunocompromised patient subgroups are more limited. Here, we report the results of a one-year assessment of immunogenicity-related responses from patients originally included in the COVAXID prospective open-label clinical trial, in which SARS-CoV-2 specific antibody titres and neutralisation responses were assessed in relation to a third and fourth vaccine dose and, in applicable cases, to SARS-CoV-2 infection (COVID-19).

## Methods

### The COVAXID clinical trial

The prospective open-label clinical trial COVAXID (clinicaltrials.gov; NCT04780659) has previously been described.^9^ In short, inclusion criteria included individuals ≥18 years old with no known history of SARS-CoV-2 infection who had either primary or secondary immunodeficiency disorders. Patients were recruited for the study during out-patient visits across various specialties at Karolinska University Hospital, Stockholm, Sweden, with the selection process being impartial to gender. The healthy control group consisted of individuals without an immunocompromised disorder and/or immunomodulatory treatment. The original clinical trial protocol was set to conclude at 6 months after the second vaccine dose. It included two vaccine doses (days 0 and 21) and immunogenicity measurements at six timepoints (days 0, 10, 21, 35 and months 3 and 6). The clinical trial was subsequently extended for a period of up to two years after the second vaccine dose. 356 of 539 (66·0%) study participants consented to continued participation in the extended clinical study, which included 2 additional timepoints, 9 and 12 months after second dose. Demographics data such as age and gender, and other medically relevant information was collected via electronic health records and national vaccination registries (Vaccinera), including medications, hospitalisation and vaccination dates. Subgroups were defined based on criteria set at the initiation of the clinical trial. Blood samples and associated clinical data were collected between February 23, 2021 and May 9, 2022. Average follow-up time was 401 days after second dose.

### Procedures

**Fig. 1:**
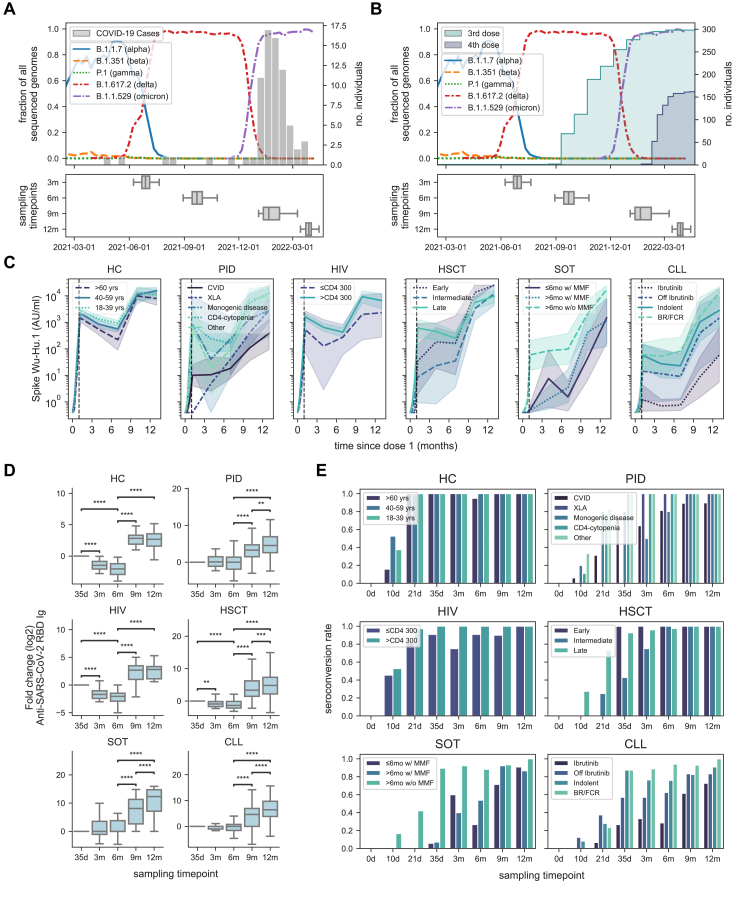
**Dynamics of antibody titres of the COVAXID cohort.** Epidemiology of prevailing SARS-CoV-2 subvariants in Sweden during the study period in relation to (A) number of verified SARS-CoV-2 infections (histogram) among study participants and (B) administration of third (shaded green cumulative histogram) and fourth (shaded blue cumulative histogram) mRNA vaccine doses. Below each graph, boxplots show the distribution of sample dates for each sampling timepoint. (C) Dynamics of Spike Ig RBD Ab titres (geometric mean with 95% CI) (shaded range) for each subgroup. The vertical dotted line represents the timepoint for the primary endpoint of the original clinical trial (35-day timepoint). (D) Fold change of Spike-RBD titres at each timepoint at a study group level. Values are normalized to the day 35-timepoint. Statistical tests were performed on paired Spike-RBD titres using Wilcoxon, and Bonferroni correction for multiple comparisons. (E) Seroconversion rates over time in each subgroup as defined by Spike RBD titres ≥0·8 AU/ml in the entire COVAXID cohort. Ab titres were quantified using the Roche-Elecsys platform. The star annotation (∗) indicates statistical significance at a p-value threshold of 0.05 (or ∗∗ for p < 0.01, ∗∗∗ for p < 0.001, ∗∗∗∗ for p < 0.0001). For sample sizes, please see Table 1. Whiskers for all box plots represents 1.5× IQR.

Serum and plasma were collected over one year and analysed for anti-SARS-CoV-2 antibody titres and neutralisation as described below. In all cases, the study subjects received monovalent mRNA vaccines according to label (BNT162b2 mRNA, Pfizer-BioNTech and in some cases mRNA-1273, Moderna) (Supplemental Table S1). The third and, in applicable cases, fourth mRNA vaccine dose was scheduled following recommendations by the Public Health Agency of Sweden. In this context, patient groups considered severely immunocompromised were offered a third vaccine dose, in most cases between months 6 and 9, and a subsequent fourth dose between months 9 and 12. In most cases, other patients and controls were offered the third dose between months 9 and 12 (Fig. 1A, Table 1). Clinical study-associated data, including verified SARS-CoV-2 infection, were recorded in an electronic case report form (eCRF). Presence of previous SARS-CoV-2 infection was recorded via in-person or phone interviews associated to each sampling timepoint. Only infections confirmed with PCR and/or rapid antigen test (RAT) was accepted as a verified SARS-CoV-2 infection. Home-testing (RAT) was initiated on the patient's own initiative. PCR tests conducted were verified through manual review of the patients' electronic health records. Additionally, patients were classified as having had a SARS-CoV-2 infection if IgG anti-nucleocapsid antibody titres were >5000 AU/ml (Meso Scale Diagnostics, MSD). Disease severity was assessed during in-person/phone interview and scored using an ordinal scale (1–8).^18^ The categories were defined as follows: 1, not hospitalized and no limitations of activities; 2, not hospitalized, with limitation of activities, home oxygen requirement, or both; 3, hospitalized, not requiring supplemental oxygen and no longer requiring ongoing medical care (used if hospitalization was extended for infection-control or other nonmedical reasons); 4, hospitalized, not requiring supplemental oxygen but re-quiring ongoing medical care (related to Covid-19 or to other medical conditions); 5, hospitalized, requiring any supplemental oxygen; 6, hospitalized, requiring non-invasive ventilation or use of high-flow oxygen devices; 7, hospitalized, receiving invasive mechanical ventilation or extracorporeal membrane oxygenation (ECMO); and 8, death.

**Table 1:** Study cohort characteristics.

Study group	HC (n = 56)			PID (n = 55)					HIV (n = 50)		HSCT (n = 52)			SOT (n = 51^a^)			CLL (n = 65)			
Subgroup	>60 yrs	40–59 yrs	18–39 yrs	CVID	XLA	Monogenic disease	CD4-cytopenia	Other	≤CD4 300	>CD4 300	Early	Intermediate	Late	≤6 mo w/MMF	>6 mo w/MMF	>6 mo w/o MMF	Ibrutinib	Off Ibrutinib	Indolent	BR/FCR
Study subjects [n]	21	19	16	33	2	5	9	6	11	39	3	8	41	17	14	20	15	8	25	17
**Demography**																				
Age in years at inclusion [median, IQR]	70 (68–75)	55 (52–57)	32 (30–34)	53 (48–62)	43 (41–45)	39 (30–41)	60 (54–73)	57 (49–64)	57 (48–67)	54 (46–68)	59 (56–64)	60 (49–63)	64 (54–69)	55 (50–61)	53 (41–69)	61 (52–70)	73 (69–76)	71 (65–75)	71 (61–77)	73 (69–75)
Gender [n, % females]	14 (67%)	10 (52%)	8 (50%)	20 (61%)	0 (0%)	4 (80%)	6 (67%)	6 (100%)	4 (36%)	18 (46%)	1 (33%)	4 (50%)	19 (46%)	5 (29%)	8 (57%)	12 (60%)	4 (27%)	3 (38%)	14 (56%)	3 (18%)
**Vaccine doses**																				
3 doses [n, %]	18 (90%)	17 (90%)	14 (88%)	30 (91%)	2 (100%)	3 (60%)	9 (100%)	4 (66%)	9 (82%)	30 (79%)	3 (100%)	8 (100%)	40 (98%)	17 (100%)	14 (100%)	20 (100%)	15 (100%)	8 (100%)	25 (100%)	14 (82%)
4 doses [n, %]	–	–	–	24 (73%)	1 (50%)	1 (20%)	6 (67%)	–	–	–	2 (67%)	8 (100%)	25 (61%)	14 (82%)	13 (93%)	15 (75%)	13 (87%)	7 (88%)	17 (68%)	12 (71%)
**Days from dose 3 to sampling**																				
9 m sampling timepoint [mean, IQR]	63 (62–74)	34 (25–46)	27 (19–37)	50 (56–82)	71 (62–80)	48 (40–60)	85 (80–96)	59 (39–86)	35 (3–60)	36 (15–53)	121 (119–122)	127 (120–139)	127 (124–136)	125 (119–138)	120 (117–136)	126 (117–145)	70 (63–82)	50 (57–66)	54 (55–75)	66 (64–78)
12 m sampling timepoint [mean, IQR]	136 (136–146)	99 (92–108)	97 (91–100)	121 (126–146)	139 (131–146)	102 (95–116)	139 (137–142)	120 (100–148)	108 (73–142)	102 (84–112)	177 (173–179)	167 (163–176)	167 (168–177)	186 (181–195)	183 (182–193)	179 (180–196)	141 (140–154)	120 (101–143)	128 (121–152)	140 (139–150)
**Days from dose 4 to sampling**																				
12 m sampling timepoint [mean, IQR]	–	–	–	26 (16–40)	38	14 (14–14)	29 (20–36)	–	–	–	38 (35–41)	26 (25–31)	35 (28–42)	41 (30–47)	37 (28–43)	36 (28–41)	28 (29–37)	25 (12–37)	27 (22–34)	27 (22–33)
**Days between vaccine doses**																				
Vaccine dose nr 3 and 4	–	–	–	115 (98–123)	116	104	112 (105–119)	–	–	–	139 (129–141)	142 (105–148)	136 (132–147)	150 (142–159)	146 (137–160)	144 (150–160)	116 (109–119)	105 (93–115)	116 (108–125)	114 (109–124)
**Seroconversion (>0.8 AU/ml)**																				
At 3 m sampling timepoint [%]	100%	100%	100%	64%	100%	50%	100%	100%	75%	100%	100%	75%	96%	60%	40%	92%	33%	57%	77%	89%
At 12 m sampling timepoint [%]	100%	100%	100%	90%	100%	100%	100%	100%	100%	100%	100%	100%	100%	91%	87%	100%	73%	83%	91%	100%
**SARS-CoV-2 Infections**																				
PCR/RAT verified COVID-19 [n, % of total]	2 (11%)	6 (32%)	8 (50%)	15 (46%)	1 (50%)	0 (0%)	1 (11%)	2 (33%)	0 (0%)	6 (16%)	2 (67%)	1 (13%)	5 (12%)	6 (35%)	3 (21%)	2 (10%)	3 (20%)	1 (13%)	7 (28%)	4 (24%)
Nucleocapsid+ (no PCR/RAT verification) COVID-19 [n, % of total]	4 (21%)	2 (11%)	2 (13%)	5 (15%)	1 (50%)	2 (40%)	2 (22%)	1 (17%)	4 (36%)	6 (16%)	1 (33%)	1 (13%)	4 (10%)	1 (6%)	0 (0%)	1 (5%)	0 (0%)	2 (25%)	0 (0%)	0 (0%)
Total COVID-19 [n, % of total]	6 (32%)	8 (42%)	10 (63%)	20 (61%)	2 (100%)	2 (40%)	3 (33%)	3 (50%)	4 (36%)	12 (32%)	3 (100%)	2 (25%)	9 (22%)	7 (41%)	3 (21%)	3 (15%)	3 (20%)	3 (38%)	7 (28%)	4 (24%)
Patients requiring hospital care due to COVID-19 [n]	–	–	–	–	1	–	–	–	–	–	–	–	1	1	1	–	–	–	2	–
**Immunoglobulin treatment**																				
Monocloncal antibodies [n]	–	–	–	·	1	–	–	–	–	–	–	–	–	1	–	–	–	–	3	1
IGRT [n]	–	–	–	21	2	–	2	–	–	–	–	–	6	–	–	–	1	1	–	2

Abbreviations: n: number, IQR: interquartile range, PID: primary immunodeficiency disorders, HIV: human immunodeficiency virus, HSCT: hematopoietic stem cell transplantation, SOT: solid organ transplantation, CLL: chronic lymphocytic leukemia, CVID, common variable immunodeficiency, XLA: X-linked agammaglobulinemia, MMF: mycophenolate mofetil, BR/FCR: Bendamustine and Rituximab/Fludarabine, Cyclophsphamide and Rituximab, IGRT: immunoglobulin replacement therapy, RAT: rapid antigen test.

a The different transplants in the SOT group (n = 51) were: 32 liver, 16 kidney, 5 kidney + pancreas.

### Antibody tests

Serum samples were initially tested for pan-Ig, including IgG, to SARS-CoV-2 Wu-Hu.1 (ancestral strain) Spike receptor-binding domain (RBD) using the quantitative Elecsys anti-SARS-CoV-2 Spike enzyme immunoassay as described (Roche Diagnostics).^9^ Samples were analysed as per clinical routine, with additional dilutions if antibody titres were above upper detection limit. Dilutions were performed to a maximum of 1:1000. Additionally plasma samples were tested for IgG binding to SARS-CoV-2 Spike Wu-Hu.1, B.1.1.7 (Alpha), B.1.351 (Beta), B.1.617/AY.4 (Delta), B.1.640.2 (IHU), and the following five Omicron variants B1.1.529/BA.1, BA.1+L452R, BA.1.1, BA.2, and BA.3, as well as IgG binding to Nucleocapsid using V-PLEX SARS-CoV-2 (Meso Scale Diagnostics, MSD). In addition, surrogate virus neutralisation against SARS-CoV-2 Spike Wu-Hu.1 and the VOCs mentioned above was measured using V-PLEX SARS-CoV-2 (Meso Scale Diagnostics, MSD) at the SciLifeLab Affinity Proteomics Unit in Uppsala, Sweden. The assays were performed according to the manufacturer's instructions using a 1:50,000 dilution. Antibody titres and neutralising capacity were expressed as arbitrary units (AU)/ml and % neutralisation, respectively. When comparing the MSD V-PLEX serology platform with the Elecsys anti-SARS-CoV-2 Spike immunoassay, a high correlation (Pearson r = 0·88, 95% CI 0.87–0.90, p < 0·001) between the two platforms was observed (Supplementary Figure S1).

### Statistical analysis

Statistical analysis was performed using Python (version 3.10.1) and the SciPy Stats library (version 1.9.2). Non-parametric tests were used for all comparative analyses since most comparisons were made within heterogeneous study groups with a relatively small sample size. To control for type I errors, we used Bonferroni post-hoc test. Comparisons between timepoints were considered dependent comparisons, while comparisons based on the number of vaccine doses and COVID-19 status were considered independent comparisons because subgroup compositions varied relative to the number of vaccine doses, in line with national vaccine recommendations at the time. Therefore, we used Wilcoxon signed-rank test for dependent comparisons and Mann–Whitney U-test for independent comparisons, both with Bonferroni correction for multiple tests. For dependent tests, missing data-pairs were excluded. In total, there were 95 missing sampling timepoints (out of 1424) in the reconsented study which corresponds 6.7%. The Spearman correlation test was used to assess correlation between antibody titres and COVID-19 severity. For Pearson correlation tests, we used Shapiro–Wilks test for normality. Geometric mean was used to display mean values of antibody titres. The statistical tests used are indicated in the figure legends. The star annotation (∗) indicates statistical significance at a p-value threshold of 0.05 (or ∗∗ for p < 0.01, ∗∗∗ for p < 0.001, ∗∗∗∗ for p < 0.0001). Additionally, non-dichotomized p-values are listed in Supplemental Table S1. In all figures with multiple comparisons, each comparison is indicated with a bracket with either “ns” or significance threshold indicated above. For box plots, whiskers represent 1.5× IQR, with the edges of the box representing the first and third quartile. Outliers are not plotted as individual plots. For subgroup analysis, individual data points are overlaid as a stripplot. Calculation of 95% confidence intervals for sensitivity and specificity analysis of nucleocapsid antibodies for annotating previous SARS-CoV-2 infection was performed using the Wilson score method.

### Ethical considerations

The study, including the extension-study, was approved by the Swedish Ethical Review Board and the Swedish Medical Products Agency (no. 2021-06046-02 and no. 5.1-2021-92151, respectively). Informed consent was obtained from all study participants prior to inclusion in the study.

### Role of funding source

The funders did not influence the study design, data collection, data analyses, interpretation, writing of the report, or decision to submit the paper for publication.

## Results

In the present study, we followed a large group of study subjects from the COVAXID clinical trial^9^ over a period of one year, in a real-world setting, with respect to anti-SARS-CoV-2 immunogenicity related responses (Fig. 1A and B). A detailed description of the respective study groups and subgroups included in the present clinical study is provided in Table 1. The reconsented COVAXID study cohort (n = 356) were generally older and had higher proportion of females, whilst antibody titres at day 35 were slightly lower in HC and slightly higher in the CLL group compared to the individuals did not reconsent for the extension of the original COVAXID clinical trial (n = 183) (Supplementary Table S2). Study subjects who consented for the extended study but who were PCR positive (n = 2) or seropositive (n = 16) at the start of the original clinical trial, or did not receive the second dose within the specified time window according to the study protocol (n = 8), were excluded from analyses. Due to the low sample size, study participants in the HC and HIV who had received four vaccine doses (n = 3), and CAR-T treated HSCT patients (n = 2), were also excluded from the analyses.

As a starting point, we assessed the temporal dynamics of anti-SARS-CoV-2 Wu-Hu.1 Spike Ig antibody titres over one year using the Elecsys anti-SARS-CoV-2 immunoassay (Fig. 1C; results from days 0 to 35 incorporated for comparison^9^). Overall, antibody titres initially peaked at day 35 and then declined until the 6-month sampling timepoint in all HC, HIV and HSCT patient groups, with the exceptions of “Early” and “Intermediate” HSCT subgroups (Fig. 1C). An increase in antibody titres was subsequently observed between 6 and 9 months in all study groups (Fig. 1C and D). An additional increase was seen in the PID, allogeneic HSCT, SOT, and CLL groups between 9 and 12 months (Fig. 1C and D). With respect to specific study subgroups, comparably low antibody titres were observed in the X-linked agammaglobulinemia (XLA) and common variable immunodeficiency (CVID) subgroups over the whole 12 months study period (Supplementary Figure S2). Similarly, in patients having undergone SOT, low antibody titres were observed in both subgroups receiving mycophenolate mofetil (MMF). In patients with CLL, low antibody titres were observed in the group treated with ibrutinib (a Bruton's tyrosine kinase inhibitor) (Fig. 1C). An in-depth comparison with respect to dynamics of antibody titres over time in the respective subgroups is shown in the appendix (Supplementary Figure S2).

Seroconversion (>0.8 AU/ml) at day 35 was the primary endpoint in the original COVAXID clinical trial.^9^ In the present follow-up, all study subjects in the healthy control group and nearly all in the HIV and allogeneic HSCT patient groups had seroconverted by the 3-month sampling timepoint and remained seroconverted throughout the study period (Table 1, Fig. 1E). Incomplete seroconversion rate at 12 months (below 95%) was seen in the PID CVID group; in the SOT subgroups with MMF; and in the CLL subgroups with ibrutinib, off ibrutinib, and early-stage untreated CLL (indolent) (Table 1).

**Fig. 2:**
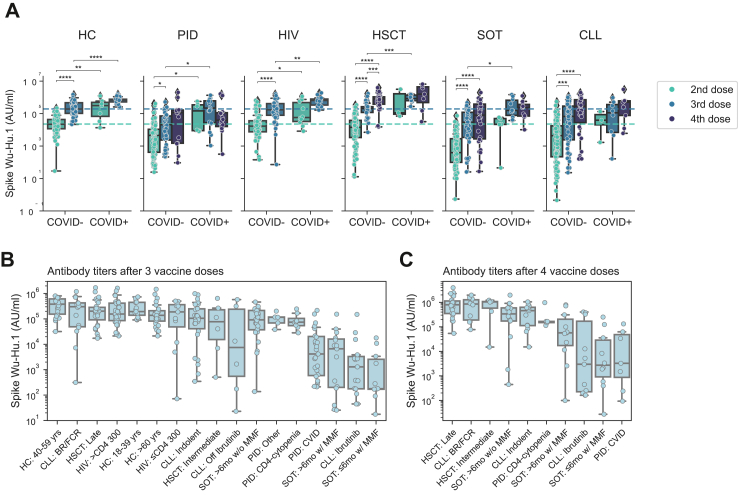
**Quantitative serological response in relation to number of vaccine doses and SARS-CoV-2 infection (COVID-19).** (A) Box plot showing Spike Wu-Hu.1 stratified based on COVID-19 status as defined by prior positive COVID-19 test (PCR/RAT) and/or anti-nucleocapsid Ab titres >5000 AU/ml. Subgroup analysis of Spike Wu-Hu.1 Ab titres following three (B) and four (C) vaccine doses ordered by the mean Ab-titre levels in each subgroup. Only subgroups with more than five recorded samples are displayed. All Ab titres were quantified using V-PLEX Serology Panels (MSD). Statistical tests performed were Mann–Whitney with Bonferroni correction for multiple comparisons. The star annotation (∗) indicates statistical significance at a p-value threshold of 0.05 (or ∗∗ for p < 0.01, ∗∗∗ for p < 0.001, ∗∗∗∗ for p < 0.0001). For sample sizes, please see Table 1. Whiskers for all boxplots represents 1.5× IQR.

In the present real-world follow-up study, the third and, in applicable cases, fourth mRNA vaccine dose was scheduled following recommendations by the Public Health Agency of Sweden. Hence, the temporal studies described above did not depict responses with respect to number of vaccine doses given and infection during the study period. Therefore, we next stratified into groups having received two, three, or four vaccine doses and, in applicable cases, infection (Fig. 2A). Focusing initially on non-infected COVID-negative (COVID-) study subjects, higher Spike antibody titres were observed in all study groups having received a third vaccine dose compared to two doses (Fig. 2A). An additional fourth dose, when provided (not provided to all groups within the present study period), yielded higher Spike antibody titres in the HSCT group (Fig. 2A). Several of the study subjects were infected (COVID-19) during the study period, in particular following the emergence of the Omicron outbreak (Fig. 1A and Table 1). Taking infection into consideration, in the COVID-positive (COVID+) study subgroups, two vaccine doses and infection yielded comparable antibody titres to three doses without a recorded infection (Fig. 2A). Overall, a higher number of vaccine doses and presence of verified infection correlated with higher antibody titres (Fig. 2A). Importantly, however, comparative analyses among the subgroups revealed large differences. Noteworthy, particularly poor responding subgroups, despite three and four vaccine doses, were identified among CVID (PID), both subgroups with MMF (SOT) and Ibrutinib (CLL) subgroups (Fig. 2B and C; Supplementary Figure S3).

**Fig. 3:**
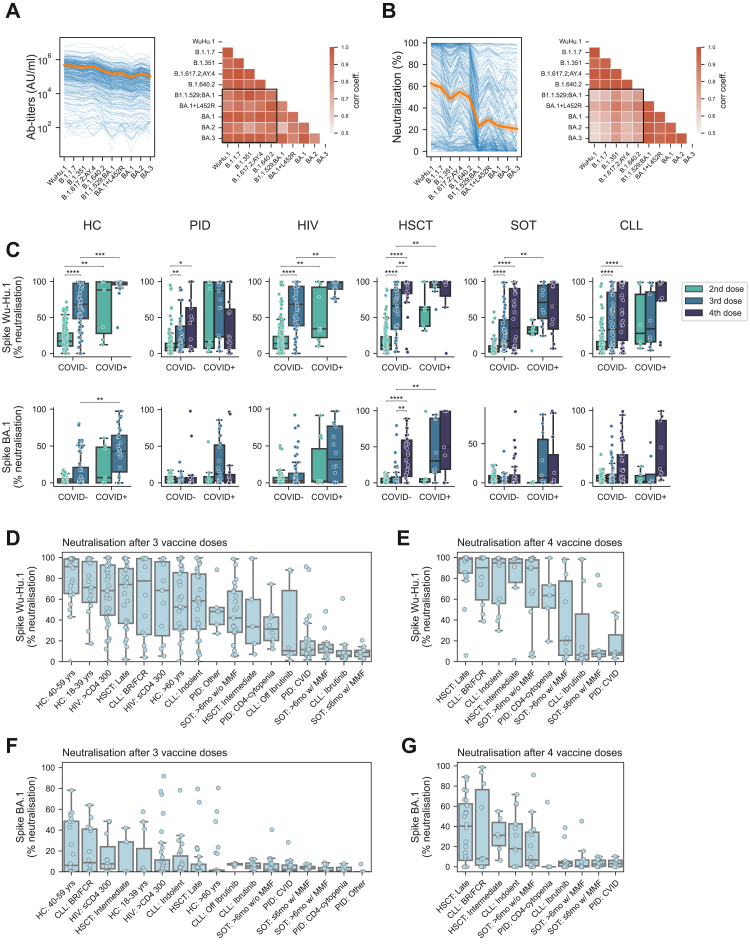
**Antibody titres and neutralising capacity of SARS-CoV-2 Omicron in relation to number of vaccine doses and SARS-CoV-2 infection (COVID-19).** Ab titres (A) and neutralizing capacity (B) of SARS-CoV-2 variants at the 12-month timepoint (left) showing individual samples as blue connected lines, and mean value across SARS-CoV-2 variants (orange line), and correlation matrices (Pearson correlation) of Ab titres for analysed SARS-CoV-2 variants (right) with correlations between Omicron-variants and non-Omicron variants in highlighted (black rectangle) areas. (C) Box plot showing neutralisation of individual samples, grouped based on COVID-19 status as defined by prior positive COVID-19 test and/or Nucleocapsid Ab titres >5000 AU/ml. Neutralisation was quantified using V-PLEX Serology Panels with an ACE2 surrogate virus neutralisation kit (Meso Scale Diagnostics, MSD). (D–G) Neutralisation of Spike Wu-Hu.1 and Spike BA.1 following three or four vaccine doses in each patient subgroup, ordered by their respective mean neutralizing capacity. Only subgroups with more than five recorded samples are displayed. Statistical tests performed were Mann–Whitney with Bonferroni correction for multiple comparisons. The star annotation (∗) indicates statistical significance at a p-value threshold of 0.05 (or ∗∗ for p < 0.01, ∗∗∗ for p < 0.001, ∗∗∗∗ for p < 0.0001). For sample sizes, please see Table 1. Whiskers for all boxplots represents 1.5× IQR.

This far, all studies described were performed with respect to antibody responses to the ancestral strain (WuHu.1). Next, we sought out to analyse binding antibody responses (titres) to all SARS-CoV-2 subvariants prevailing during the study period. Irrespective of assessed subvariant, overall strikingly similar antibody titres were observed with respect to reactivity towards all SARS-CoV-2 Spike subvariant studied (Fig. 3A, left), yielding a high degree of correlation in comparative analysis (Fig. 3A, right). This led us to further analyse neutralizing antibody responses to all SARS-CoV-2 subvariants prevailing during the study period. Here, markedly different antibody neutralisation responses were observed between non-Omicron and Omicron variants (Fig. 3B, left), yielding a low correlation coefficient between non-Omicron and Omicron variants (Fig. 3B, right). This in turn prompted us to address neutralizing responses in more detail, focusing on comparative studies between neutralisation against WuHu.1 and the Omicron BA.1 variant. A significant increase of WuHu.1-specific neutralisation was seen following the third vaccine dose in all major study groups (Fig. 3C, upper panel). However, the response from a third dose was more or less absent with respect to Omicron BA.1-specific neutralisation (Fig. 3C, lower panel). A fourth dose, or the addition of a recorded SARS-CoV-2 infection was associated with increased Omicron BA.1-specific neutralisation in several of the study groups (Fig. 3C, lower panel). When the results were addressed at a subgroup level, the findings above with strikingly poor responses against Omicron BA.1 compared to Wu-Hu.1 was recapitulated in all subgroups following three vaccine doses (compare Fig. 3D and F; Supplementary Figure S4). Following a fourth vaccine dose, Omicron responses were increased in several subgroups while remaining low in others, including in the Ibrutinib (CLL) subgroup, in both subgroups with MMF (SOT), and in the CVID (PID) subgroup (Fig. 3E and G; Supplementary Figure S4). In conclusion, these results highlight significantly poor vaccine-elicited neutralising responses towards Omicron subvariants in specific subgroups within the present study cohort.

**Fig. 4:**
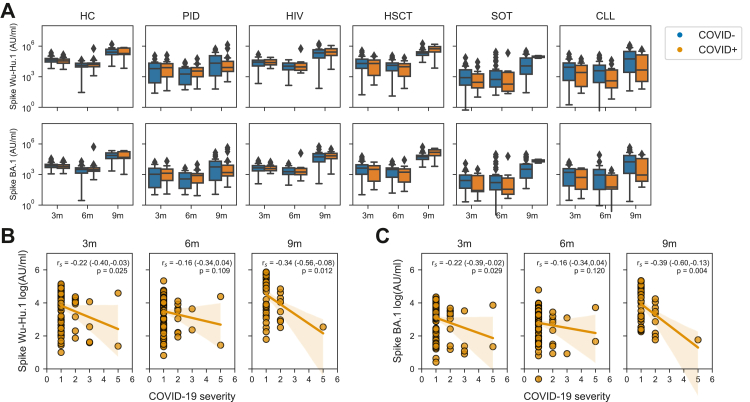
**Prospective risk for SARS-CoV-2 infection and COVID-19 severity in relation to antibody titres.** (A) Ab titres at 3-, 6-, and 9-months in patients without a prior history of COVID-19 (and Nucleocapsid Ab titres <5000 AU/ml) in relation to later development of COVID-19. Statistical test performed were Mann–Whitney U test. Correlation between Wu-Hu.1 (B) or BA.1 (C) reactive Ab titres at different sampling timepoints and future COVID-19 severity. Statistical tests performed were Spearman correlation. For sample sizes, please see Table 1. Whiskers for all boxplots represents 1.5× IQR.

Finally, while the present study was specifically designed to assess immunogenicity-related parameters in relation to vaccination and infection, we also assessed parameters relating to risk of infection and severe disease. More specifically, we analysed antibody titres in study subjects without a prior record of infection at different sampling timepoints. Study subjects who were later infected, did not have significantly lower antibody titres compared to those who were not infected (Fig. 4A). Furthermore, we also correlated antibody titres with COVID-19 severity among those who were infected. In this context, six of the 75 study subjects who had PCR/RAT verified COVID-19 were hospitalized (COVID-19 severity ≥3). Five of the six study subjects were infected following the Omicron outbreak. A tendency towards lower antibody titres in higher severity scores was observed (Fig. 4B and C). Since the current study was underpowered for, and not intended to investigate the prospective risk of infection and disease severity, we choose not to make any strong conclusions based on these findings.

## Discussion

Longitudinal clinical studies addressing immunity in large groups of immunocompromised patients in a real-world setting, taking vaccination as well as infection into consideration, are scarce. Such studies serve the purpose of addressing the dynamics (temporal) of antibody binding titres as well as neutralizing titres in a comparative fashion among different immunocompromised patient groups and subgroups. Here, we report one-year results from such a study, based on a study cohort derived from the original open-label, prospective, clinical trial COVAXID. The results reveal that most of the immunocompromised patient subgroups who responded poorly following the initial two doses of the BNT162b2 mRNA vaccine develop significant binding antibody titres against both the Wu-Hu.1 ancestral strain and Omicron variants following subsequent booster vaccine doses, and in applicable cases SARS-CoV-2 infection. However, while several of the study groups developed neutralizing antibody responses towards the ancestral strain, markedly lower responses were observed towards Omicron subvariants. Notably, Omicron-specific neutralisation increased after a fourth dose as well as after three doses and one infection in many of the patient groups.

In terms of long-term antibody responses to SARS-CoV-2 mRNA vaccination, our data grossly identifies three classes of immunocompromised patients broadly defined by their serological response patterns; 1) strong responders, e.g., patients having undergone HSCT and PLWH, 2) poor responders, e.g., patients having undergone SOT and treated with MMF, patients with CVID, and patients with CLL treated with ibrutinib, and 3) non-responders, e.g., patients with XLA. The “strong responders” showed responses equivalent to healthy controls over time. Although patients having undergone HSCT are still recommended to follow certain health-related precautions, we did not observe a compromised long-term antibody response following repeated vaccinations. The latter, most likely due to their reconstituted immune system over time. The “poor responders” comprise of a diverse group of patients. Many of the poorest responders were patients undergoing concurrent immunosuppressive treatment during vaccination. Some of these patient groups may benefit from repeated and/or more frequent vaccinations. The “non-responders” are incapable of responding with antibody responses due do their underlying disease. Importantly, some of these patients have been shown to generate strong T-cell mediated response following vaccination.^19^^,^^20^

Notably, study participants included in the present real-world study were subject to necessary clinical interventions due to their specific medical conditions. The latter has included therapeutic interventions with immunoglobulin replacement therapy (IGRT), affecting antibody titres in some patient groups (Table 1). In this respect, newer batches of IGRTs contain antibodies against the SARS-CoV-2 Spike and, to a lower degree, Nucleocapsid antigens.^21^^,^^22^ These antibodies likely contributed to observed antibody titres, and corresponding seroconversion, over time in some of these groups, e.g., the CVID and XLA groups (Fig. 1 and Table 1). Likewise, a few patients from the study groups were treated with anti-SARS-CoV-2 monoclonal antibodies (Table 1). The number of infections in the different groups after vaccination did not differ significantly. However, the number of serious covid-infections were very few across the groups, underscoring the fact that vaccination was overall efficient in preventing severe disease, whereas the protection against mild disease with omicron variants was rather poor. However, risk for infection also correlates with exposure. In this context many of the present patient groups take precautionary measure due to their underlying disease.

A strength of the present study is that data were analysed from a well-defined patient cohort based on the original clinical trial COVAXID, with set inclusion and exclusion criteria as well as independent study monitoring of data, all of which contributed to data quality. Another strength of the study is that patient groups were followed from a timepoint when they were SARS-CoV-2- and vaccination-naïve through the emergence of updated national vaccination schedule regimens and in parallel risk for exposure to multiple new SARS-CoV-2 variants, all of which together provided the possibility for a complete and comprehensive longitudinal serological assessment of the study cohort in a real-world setting. The number and frequency of study participant samplings, as well as the ability to directly compare results across subgroups and corresponding different immunodeficiency disorders, provided additional strength to the study. With respect to limitations, as mentioned the individual patients had access to the third and fourth vaccine doses based on priority as recommended by the Public Health Agency of Sweden, i.e., not as part of a pre-defined clinical schedule. Furthermore, while most patients received the mRNA BNT162b2 (Pfizer/BioNTech) vaccine, some received the mRNA1273 (Moderna) vaccine for their third and/or fourth doses (Supplementary Table S3). In this respect, studies have observed slightly better serological responses with the mRNA1273 vaccine than with the BNT162b2 mRNA vaccine.^23^ With respect to COVID-19, the diagnosis annotated to patients in the study groups which were PCR or rapid antigen test (RAT) positive, and/or had presence of anti-nucleocapsid antibodies (>5000 AU/ml). The sensitivity and specificity of anti-nucleocapsid antibodies for PCR/RAT verified COVID-19 in our cohort was 0.44 (0.36, 0.52) and 0.95 (0.94, 0.96), respectively (Supplementary Figure S5), which could predispose for false-negative COVID-19 annotations. Furthermore, anti-nucleocapsid antibody titres may occur in patients who have received IGRT which could predispose for false-positive COVID-19 annotations. An additional limitation of our study pertains to unaddressed confounding factors potentially affecting measured antibody titres. Specifically, variables such as the age, gender and timing, type, and dosage of immunosuppressive treatment administered during the follow-up period were not adjusted for in the antibody titre measurements due to the limited sizes of the subgroups in the current study.

In conclusion, the present results provide a detailed comparative assessment of immunogenicity in several groups of immunocompromised patients following the third and fourth doses of mRNA vaccine as well as the result of SARS-CoV-2 infection in a real-world setting. The overall results should be generalizable to similar patient groups at other sites, in part since they mimic findings from other studies published. Proactive measures with continuously repeated vaccinations in vulnerable patient groups, despite seroconversion and significant antibody titres against the presently dominant virus strain, may still be beneficial as it could provide a degree of cross-reactivity in case of substantial mutations in future SARS-CoV-2 variants. Additionally, individuals with immunocompromising disorders might benefit from updated vaccines that target Omicron new subvariants and possible future new emerging SARS-CoV-2 variants-of-concerns. Taken together, the present data add additional information serving to improve the management of immunocompromised patients, many of which represent risk groups for severe COVID-19.

## Contributors

PB, OB, LH, SM, PN, GS, AÖ, CIES, KL, MSC, MB, PL, SA, and HGL contributed to conceptualization, funding acquisition and discussion of data. PL and SA wrote the original clinical trial protocol. PB, LH, SM, PN, GS and SA functioned as primary investigator for each patient study group, including recruitment of study participants and clinical management of study participants during the trial. OB, AÖ and JV recruited study participants and were co-responsible for clinical management of study participants during the trial. PB, LH, SM, PN, GS and SA have collected and curated clinical data. PC, PB, LH, SM, PN, GS, PL, SA and HGL were responsible for project administration. PC, MA, DW, AC and MÅ contributed to project administration through planning and coordinating sample collection and associated data collection. GB, SMu and MÅ contributed to investigation through sample analyses. SA and HGL contributed to project administration, resources, and supervision of the trial. PC and SA have had access, and verified all underlying data reported in the manuscript. PC and HGL contributed to visualization and analysis of data. PC and HGL wrote the first draft of the manuscript, with input from PB, OB, LH, SM, PN, GS, AÖ, CIES, JV, DW, AC, MÅ, KL, MSC, MB, PL, and SA. All authors read and approved the manuscript prior to submission.

## Data sharing statement

Relevant data will be submitted to European Union Drug Regulating Authorities Clinical Trials Database (EudraCT). The full original clinical study protocol is available via the SciLifeLab Data Repository (English version: https://doi.org/10.17044/scilifelab.15059364; Swedish version https://doi.org/10.17044/scilifelab.15059355). Anonymous data displayed in the manuscript will be made available upon request to the corresponding author following publication of the present article. Data displayed in the manuscript or acquired during the clinical trial, will be made available in a form not deviating from what is accepted by local regulatory authorities with respect to handling of patient data, and in adherence of the policies of the Karolinska University Hospital and Karolinska Institutet.

## Declaration of interests

PB has received honoraria from Takeda for educational lectures not directly relevant to this work. SM has received honoraria from Celgene/BMS, Novartis, Gilead/Kite, DNA Prime for lectures and educational events and as a member and/or head of data safety monitoring boards from Miltenyi and Immunicum not directly relevant to this work. CIES has received financial support from Moderna for work not directly relevant to this work. KL has received financial support from Moderna for work not directly relevant to this work. PL has received grants from Pfizer, MSD, and personal fees from Takeda, AiCuris, and OctaPharma, not directly relevant to the submitted work. SA has received honoraria for lectures and educational events, from Gilead, AbbVie, MSD, Biogen and Netdoktor, not directly related to this work, and reports grants from the Swedish Research Council on COVID-19 vaccination. HGL received honoraria from Sanofi for consultation not relevant to this work, and has served on the UK-CIC Oversight Committee, had led the Karolinska Institutet COVID-19 vaccine group, and is on the scientific advisory group for the International Vaccine Institute not directly relevant to this work, and reports grants from Knut and Alice Wallenberg Foundation, Nordstjernan AB, Region Stockholm, and Karolinska Institutet for studies on COVID-19 and COVID-19 vaccination. All other authors declare no potential or actual conflict of interest to the work presented in this paper.
