# Supplementary material for: Real-world assessment of immunogenicity in immunocompromised individuals following SARS-CoV-2 mRNA vaccination: a one-year follow-up of the prospective clinical trial COVAXID
Source: eBioMedicine. 2023 Jul 13;94:104700. doi: 10.1016/j.ebiom.2023.104700 (PMC10365982; doi:10.1016/j.ebiom.2023.104700)
Supplement: Supplementary Figures S1–S5 and Tables S1–S3 [file mmc1.pdf]

Appendix: Supplemental Figures & Tables

Supplement to: Chen P, Bergman P, Blennow O, et al. Real-world assessment of immunogenicity in immunocompromised individuals following SARS-CoV-2 mRNA vaccination: A one-year follow-up of the prospective clinical trial COVAXID

Table of contents

Figures

p1. Supplementary Figure S1: Correlation between Roche-Elecsys and MSD platform for SARS-CoV-2 Spike-RBD

p2. Supplementary Figure S2: Subgroup-stratified dynamics of SARS-CoV-2 Wu-Hu.1 Ab titres

p3. Supplementary Figure S3: Comparison of Ab titres between subgroups

p4. Supplementary Figure S4: Comparison of Spike neutralization between subgroups

p5. Supplementary Figure S5: Nucleocapsid titres in verified and unverified COVID-19 cases

Tables

p6. Supplementary Table S1: Non-dichotomized p-values

p15. Supplementary Table S2: Demographics and antibody titres in reconsented study subjects and drop-outs

p16. Supplementary Table S3: Number of administered 3rd and 4th vaccine doses

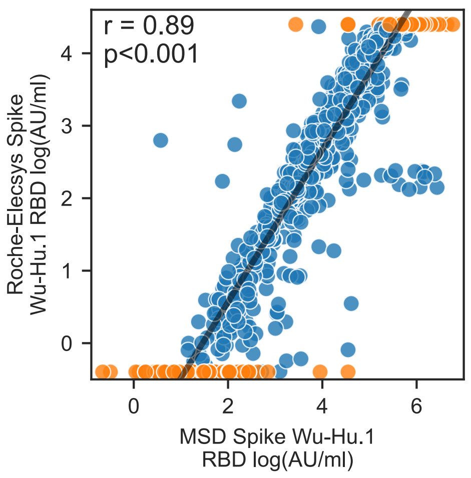

Supplementary Figure S1. Correlation between Roche-Elecsys and MSD platform for SARS-CoV-2 Spike-RBD. Values below 0·4 or above 25,000 on the Elecsys platform are indicated in orange. R = Pearson correlation coefficient. Correlation and p-value are calculated on log-transformed values within detection range on the Roche-Elecsys platform.

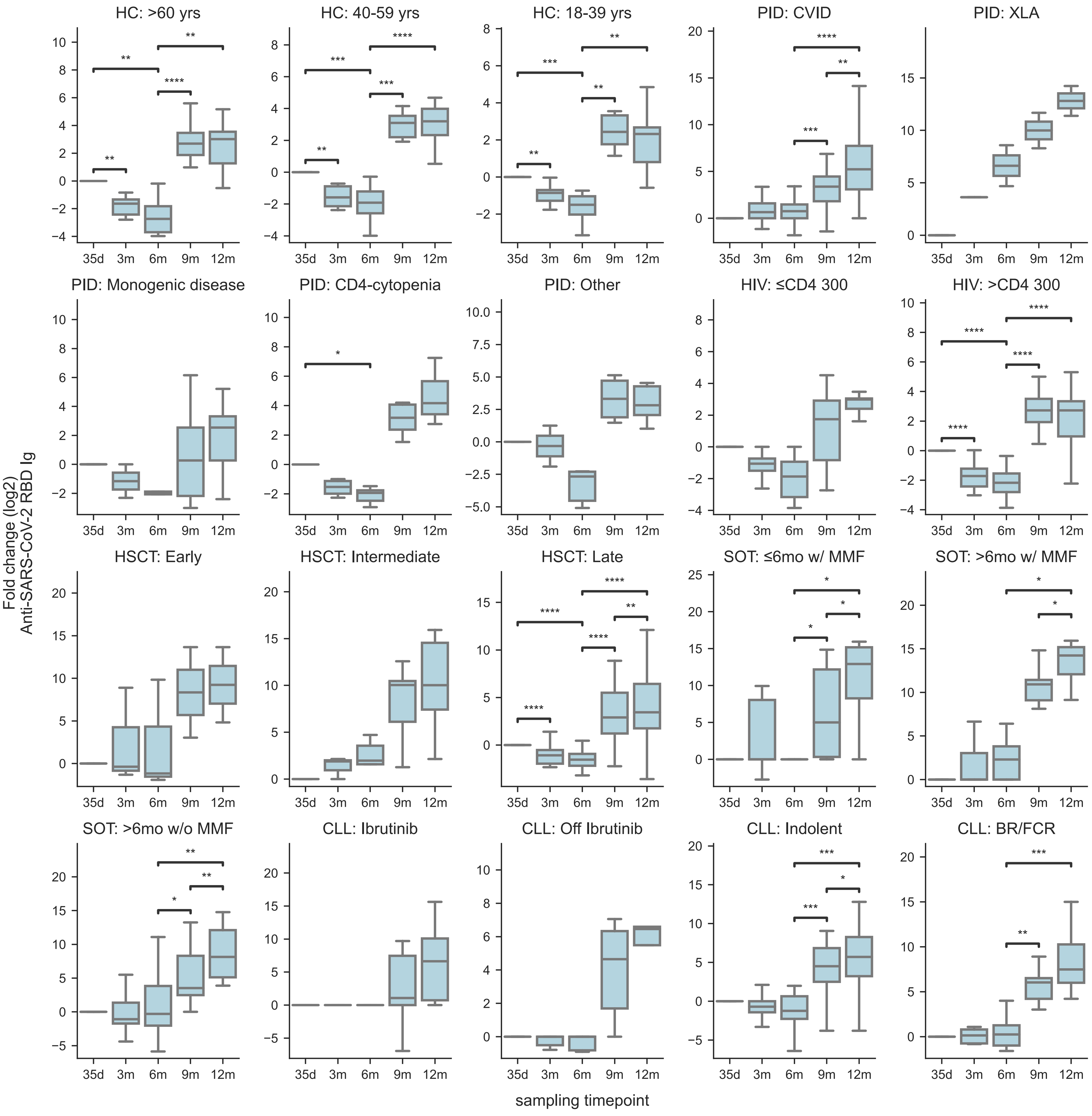

Supplementary Figure S2. Subgroup-stratified dynamics of SARS-CoV-2 Wu-Hu.1 Ab titres. Fold change of Spike-RBD titres at each timepoint at a subgroup level. Values are normalized to the day 35-timepoint. Statistical tests were performed on paired Spike-RBD titres using Wilcoxon, and Bonferroni correction for multiple comparisons. The star annotation (*) indicates statistical significance at a p-value threshold of 0.05 (or ** for $p < 0.01$, *** for $p < 0.001$, **** for $p < 0.0001$). For sample sizes, please see Table 1. Whiskers for all boxplots represents 1.5x IQR.

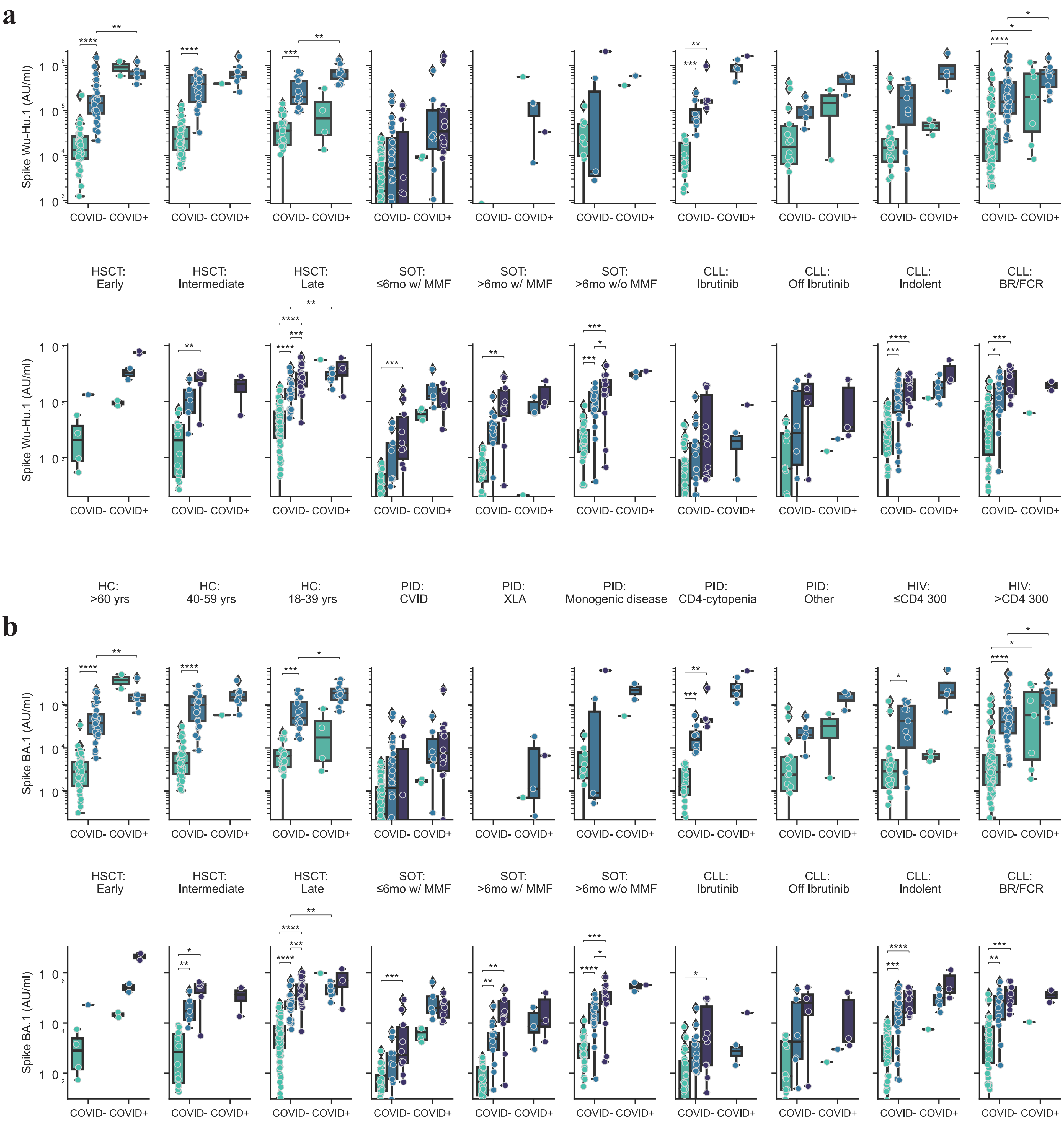

Supplementary Figure S3. Comparison of Ab titres between subgroups. Box plot showing (A) Spike Wu-Hu.1 and (B) Spike BA.1 Ab titres, stratified based on COVID-19 status as defined by prior positive COVID-19 test (PCR/RAT) and/or Nucleocapsid Ab titres >5,000 AU/ml in each patient subgroup. Statistical tests performed were Mann-Whitney with Bonferroni correction for multiple comparisons. The star annotation (*) indicates statistical significance at a p-value threshold of 0.05 (or ** for $p < 0.01$, *** for $p < 0.001$, **** for $p < 0.0001$). For sample sizes, please see Table 1. Whiskers for all boxplots represents 1.5x IQR.

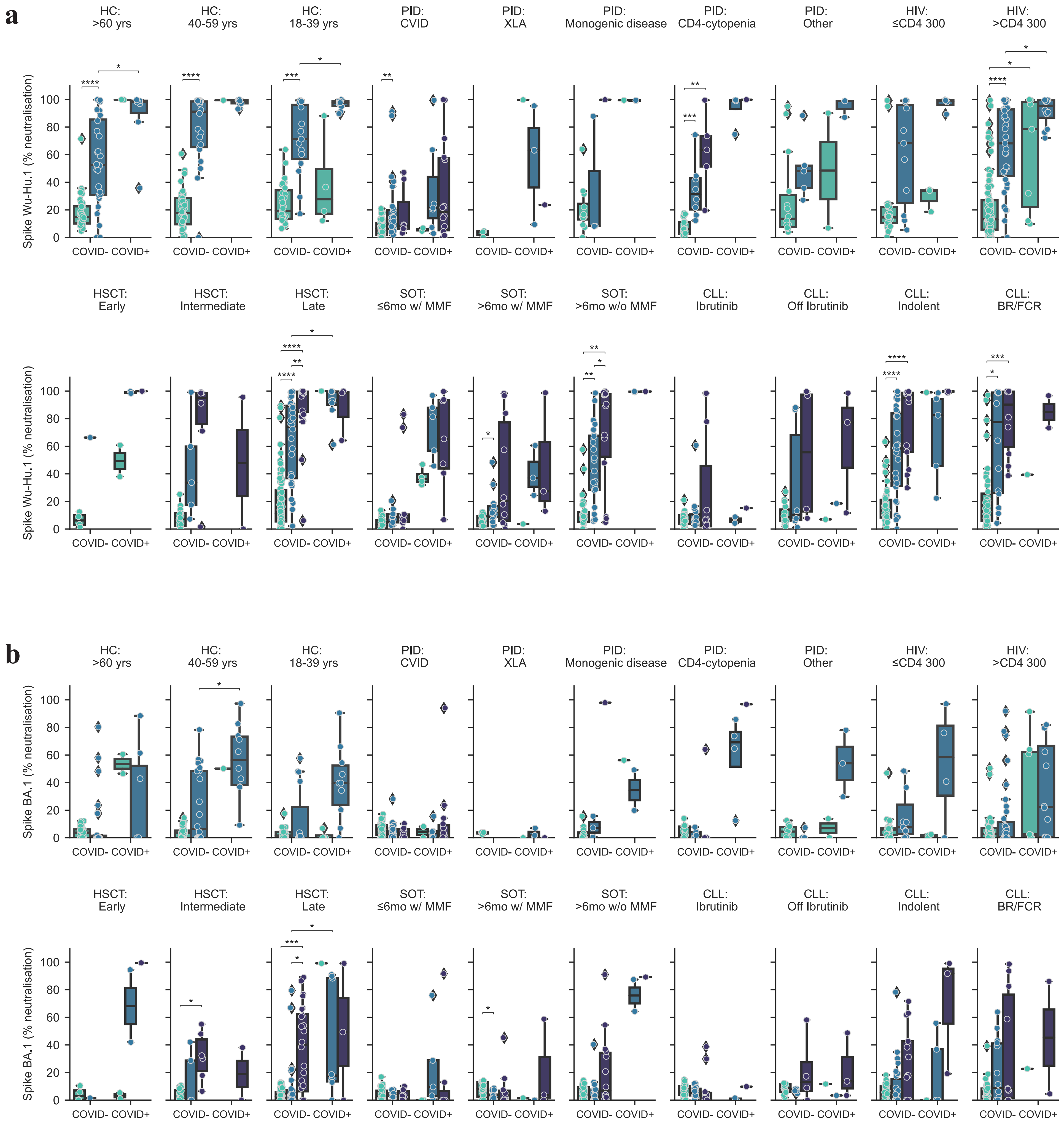

Supplementary Figure S4. Comparison of Spike neutralisation between subgroups. Box plot showing Ab neutralisation against Wu-Hu.1 (A) and BA.1 (B), stratified based on COVID-19 status as defined by prior positive COVID-19 test (PCR/RAT) and/or Nucleocapsid Ab titres >5,000 AU/ml in each patient subgroup. Statistical tests performed were Mann-Whitney with Bonferroni correction for multiple comparisons. The star annotation (*) indicates statistical significance at a p-value threshold of 0.05 (or ** for $p < 0.01$, *** for $p < 0.001$, **** for $p < 0.0001$). For sample sizes, please see Table 1. Whiskers for all boxplots represents 1.5x IQR.

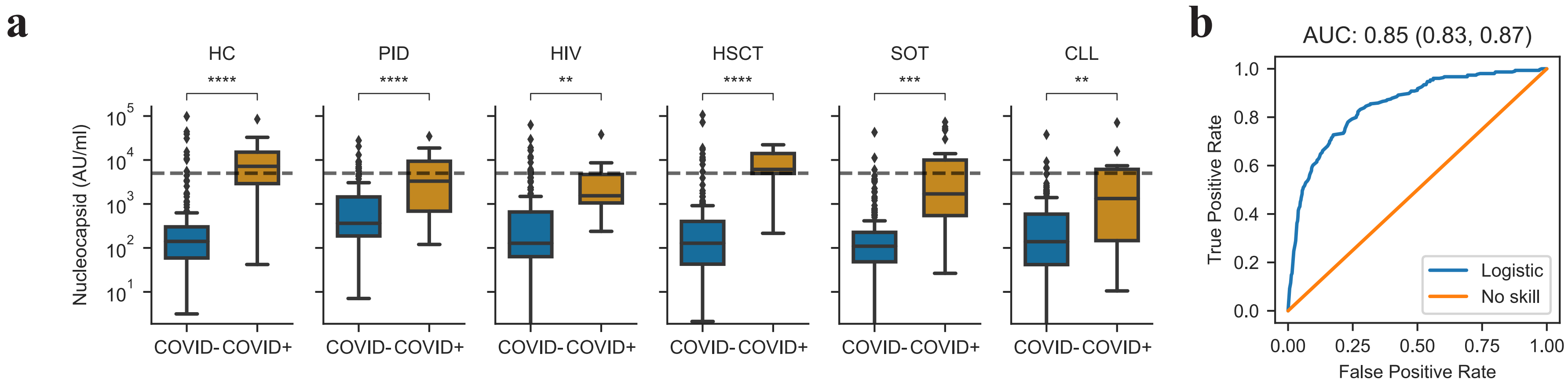

Supplementary Figure S5. Nucleocapsid titres in verified and unverified COVID-19 cases. (a) Box plot showing Nucleocapsid Ab titres stratified based on verified COVID-19 diagnosis in healthy controls and patient groups. The dotted line represents the cut-off value for COVID-19 positive (not PCR or rapid antigen test) determined by Nucleocapsid titre levels, as provided by MSD. Statistical tests were performed using Mann-Whitney U test. **(b)** Receiver operator characteristics of Nucleocapsid and COVID-19 diagnosis. The ROC curve was generated using sensitivity and specificity values calculated at different cut-off values using logistic regression, with the area under the curve (AUC, 95% CI) indicating the overall diagnostic performance. The star annotation (*) indicates statistical significance at a p-value threshold of 0.05 (or ** for $p < 0.01$, *** for $p < 0.001$, **** for $p < 0.0001$). For sample sizes, please see Table 1. Whiskers for all boxplots represents 1.5x IQR.

Figure	Group	Variant	Comparison A	Comparison B	p-value
**Fig 1b**	HC	WuHu.1	35d	3m	<0·0001
**Fig 1b**	HC	WuHu.1	35d	6m	<0·0001
**Fig 1b**	HC	WuHu.1	6m	9m	<0·0001
**Fig 1b**	HC	WuHu.1	6m	12m	<0·0001
**Fig 1b**	HC	WuHu.1	9m	12m	1·00
**Fig 1b**	PID	WuHu.1	35d	3m	1·00
**Fig 1b**	PID	WuHu.1	35d	6m	0·056
**Fig 1b**	PID	WuHu.1	6m	9m	<0·0001
**Fig 1b**	PID	WuHu.1	6m	12m	<0·0001
**Fig 1b**	PID	WuHu.1	9m	12m	0·0028
**Fig 1b**	HIV	WuHu.1	35d	3m	<0·0001
**Fig 1b**	HIV	WuHu.1	35d	6m	<0·0001
**Fig 1b**	HIV	WuHu.1	6m	9m	<0·0001
**Fig 1b**	HIV	WuHu.1	6m	12m	<0·0001
**Fig 1b**	HIV	WuHu.1	9m	12m	0·64
**Fig 1b**	HSCT	WuHu.1	35d	3m	0·0017
**Fig 1b**	HSCT	WuHu.1	35d	6m	<0·0001
**Fig 1b**	HSCT	WuHu.1	6m	9m	<0·0001
**Fig 1b**	HSCT	WuHu.1	6m	12m	<0·0001
**Fig 1b**	HSCT	WuHu.1	9m	12m	0·0002
**Fig 1b**	SOT	WuHu.1	35d	3m	1·00
**Fig 1b**	SOT	WuHu.1	35d	6m	1·00
**Fig 1b**	SOT	WuHu.1	6m	9m	<0·0001
**Fig 1b**	SOT	WuHu.1	6m	12m	<0·0001
**Fig 1b**	SOT	WuHu.1	9m	12m	<0·0001
**Fig 1b**	CLL	WuHu.1	35d	3m	0·51
**Fig 1b**	CLL	WuHu.1	35d	6m	1·00
**Fig 1b**	CLL	WuHu.1	6m	9m	<0·0001
**Fig 1b**	CLL	WuHu.1	6m	12m	<0·0001
**Fig 1b**	CLL	WuHu.1	9m	12m	<0·0001
**Fig 2a**	HC	WuHu.1	(COVID-, 2 doses)	(COVID-, 3 doses)	<0·0001
**Fig 2a**	HC	WuHu.1	(COVID+, 2 doses)	(COVID+, 3 doses)	0·30
**Fig 2a**	HC	WuHu.1	(COVID-, 2 doses)	(COVID+, 2 doses)	0·0013
**Fig 2a**	HC	WuHu.1	(COVID-, 3 doses)	(COVID+, 3 doses)	<0·0001
**Fig 2a**	PID	WuHu.1	(COVID-, 2 doses)	(COVID-, 3 doses)	0·022
**Fig 2a**	PID	WuHu.1	(COVID-, 2 doses)	(COVID-, 4 doses)	0·16
**Fig 2a**	PID	WuHu.1	(COVID-, 3 doses)	(COVID-, 4 doses)	1·00
**Fig 2a**	PID	WuHu.1	(COVID+, 2 doses)	(COVID+, 3 doses)	1·00
**Fig 2a**	PID	WuHu.1	(COVID+, 2 doses)	(COVID+, 4 doses)	1·00
**Fig 2a**	PID	WuHu.1	(COVID+, 3 doses)	(COVID+, 4 doses)	1·00
**Fig 2a**	PID	WuHu.1	(COVID-, 2 doses)	(COVID+, 2 doses)	0·041
**Fig 2a**	PID	WuHu.1	(COVID-, 3 doses)	(COVID+, 3 doses)	0·027
**Fig 2a**	PID	WuHu.1	(COVID-, 4 doses)	(COVID+, 4 doses)	1·00
**Fig 2a**	HIV	WuHu.1	(COVID-, 2 doses)	(COVID-, 3 doses)	<0·0001
**Fig 2a**	HIV	WuHu.1	(COVID+, 2 doses)	(COVID+, 3 doses)	0·23
**Fig 2a**	HIV	WuHu.1	(COVID-, 2 doses)	(COVID+, 2 doses)	0·013
**Fig 2a**	HIV	WuHu.1	(COVID-, 3 doses)	(COVID+, 3 doses)	0·0034
**Fig 2a**	HSCT	WuHu.1	(COVID-, 2 doses)	(COVID-, 3 doses)	<0·0001
**Fig 2a**	HSCT	WuHu.1	(COVID-, 2 doses)	(COVID-, 4 doses)	<0·0001
**Fig 2a**	HSCT	WuHu.1	(COVID-, 3 doses)	(COVID-, 4 doses)	0·0002
**Fig 2a**	HSCT	WuHu.1	(COVID+, 2 doses)	(COVID+, 3 doses)	1·00
**Fig 2a**	HSCT	WuHu.1	(COVID+, 2 doses)	(COVID+, 4 doses)	1·00
**Fig 2a**	HSCT	WuHu.1	(COVID+, 3 doses)	(COVID+, 4 doses)	1·00
**Fig 2a**	HSCT	WuHu.1	(COVID-, 2 doses)	(COVID+, 2 doses)	0·16

**Fig 2a**	HSCT	WuHu.1	(COVID-, 3 doses)	(COVID+, 3 doses)	0·0008
**Fig 2a**	HSCT	WuHu.1	(COVID-, 4 doses)	(COVID+, 4 doses)	1·00
**Fig 2a**	SOT	WuHu.1	(COVID-, 2 doses)	(COVID-, 3 doses)	<0·0001
**Fig 2a**	SOT	WuHu.1	(COVID-, 2 doses)	(COVID-, 4 doses)	<0·0001
**Fig 2a**	SOT	WuHu.1	(COVID-, 3 doses)	(COVID-, 4 doses)	0·074
**Fig 2a**	SOT	WuHu.1	(COVID+, 2 doses)	(COVID+, 3 doses)	0·33
**Fig 2a**	SOT	WuHu.1	(COVID+, 2 doses)	(COVID+, 4 doses)	0·25
**Fig 2a**	SOT	WuHu.1	(COVID+, 3 doses)	(COVID+, 4 doses)	1·00
**Fig 2a**	SOT	WuHu.1	(COVID-, 2 doses)	(COVID+, 2 doses)	1·00
**Fig 2a**	SOT	WuHu.1	(COVID-, 3 doses)	(COVID+, 3 doses)	0·042
**Fig 2a**	SOT	WuHu.1	(COVID-, 4 doses)	(COVID+, 4 doses)	1·00
**Fig 2a**	CLL	WuHu.1	(COVID-, 2 doses)	(COVID-, 3 doses)	0·0001
**Fig 2a**	CLL	WuHu.1	(COVID-, 2 doses)	(COVID-, 4 doses)	<0·0001
**Fig 2a**	CLL	WuHu.1	(COVID-, 3 doses)	(COVID-, 4 doses)	0·12
**Fig 2a**	CLL	WuHu.1	(COVID+, 2 doses)	(COVID+, 3 doses)	1·00
**Fig 2a**	CLL	WuHu.1	(COVID+, 2 doses)	(COVID+, 4 doses)	1·00
**Fig 2a**	CLL	WuHu.1	(COVID+, 3 doses)	(COVID+, 4 doses)	1·00
**Fig 2a**	CLL	WuHu.1	(COVID-, 2 doses)	(COVID+, 2 doses)	1·00
**Fig 2a**	CLL	WuHu.1	(COVID-, 3 doses)	(COVID+, 3 doses)	1·00
**Fig 2a**	CLL	WuHu.1	(COVID-, 4 doses)	(COVID+, 4 doses)	1·00
**Fig 3c**	HC	WuHu.1	(COVID-, 2 doses)	(COVID-, 3 doses)	<0·0001
**Fig 3c**	HC	WuHu.1	(COVID+, 2 doses)	(COVID+, 3 doses)	1·00
**Fig 3c**	HC	WuHu.1	(COVID-, 2 doses)	(COVID+, 2 doses)	0·0040
**Fig 3c**	HC	WuHu.1	(COVID-, 3 doses)	(COVID+, 3 doses)	0·0002
**Fig 3c**	PID	WuHu.1	(COVID-, 2 doses)	(COVID-, 3 doses)	0·0015
**Fig 3c**	PID	WuHu.1	(COVID-, 2 doses)	(COVID-, 4 doses)	0·024
**Fig 3c**	PID	WuHu.1	(COVID-, 3 doses)	(COVID-, 4 doses)	1·00
**Fig 3c**	PID	WuHu.1	(COVID+, 2 doses)	(COVID+, 3 doses)	1·00
**Fig 3c**	PID	WuHu.1	(COVID+, 2 doses)	(COVID+, 4 doses)	1·00
**Fig 3c**	PID	WuHu.1	(COVID+, 3 doses)	(COVID+, 4 doses)	1·00
**Fig 3c**	PID	WuHu.1	(COVID-, 2 doses)	(COVID+, 2 doses)	0·57
**Fig 3c**	PID	WuHu.1	(COVID-, 3 doses)	(COVID+, 3 doses)	0·064
**Fig 3c**	PID	WuHu.1	(COVID-, 4 doses)	(COVID+, 4 doses)	1·00
**Fig 3c**	HIV	WuHu.1	(COVID-, 2 doses)	(COVID-, 3 doses)	<0·0001
**Fig 3c**	HIV	WuHu.1	(COVID+, 2 doses)	(COVID+, 3 doses)	0·73
**Fig 3c**	HIV	WuHu.1	(COVID-, 2 doses)	(COVID+, 2 doses)	0·0018
**Fig 3c**	HIV	WuHu.1	(COVID-, 3 doses)	(COVID+, 3 doses)	0·0036
**Fig 3c**	HSCT	WuHu.1	(COVID-, 2 doses)	(COVID-, 3 doses)	<0·0001
**Fig 3c**	HSCT	WuHu.1	(COVID-, 2 doses)	(COVID-, 4 doses)	<0·0001
**Fig 3c**	HSCT	WuHu.1	(COVID-, 3 doses)	(COVID-, 4 doses)	0·0013
**Fig 3c**	HSCT	WuHu.1	(COVID+, 2 doses)	(COVID+, 3 doses)	1·00
**Fig 3c**	HSCT	WuHu.1	(COVID+, 2 doses)	(COVID+, 4 doses)	1·00
**Fig 3c**	HSCT	WuHu.1	(COVID+, 3 doses)	(COVID+, 4 doses)	1·00
**Fig 3c**	HSCT	WuHu.1	(COVID-, 2 doses)	(COVID+, 2 doses)	0·068
**Fig 3c**	HSCT	WuHu.1	(COVID-, 3 doses)	(COVID+, 3 doses)	0·0087
**Fig 3c**	HSCT	WuHu.1	(COVID-, 4 doses)	(COVID+, 4 doses)	1·00
**Fig 3c**	SOT	WuHu.1	(COVID-, 2 doses)	(COVID-, 3 doses)	<0·0001
**Fig 3c**	SOT	WuHu.1	(COVID-, 2 doses)	(COVID-, 4 doses)	<0·0001
**Fig 3c**	SOT	WuHu.1	(COVID-, 3 doses)	(COVID-, 4 doses)	0·75
**Fig 3c**	SOT	WuHu.1	(COVID+, 2 doses)	(COVID+, 3 doses)	0·30
**Fig 3c**	SOT	WuHu.1	(COVID+, 2 doses)	(COVID+, 4 doses)	1·00
**Fig 3c**	SOT	WuHu.1	(COVID+, 3 doses)	(COVID+, 4 doses)	1·00
**Fig 3c**	SOT	WuHu.1	(COVID-, 2 doses)	(COVID+, 2 doses)	0·35
**Fig 3c**	SOT	WuHu.1	(COVID-, 3 doses)	(COVID+, 3 doses)	0·0066
**Fig 3c**	SOT	WuHu.1	(COVID-, 4 doses)	(COVID+, 4 doses)	1·00
**Fig 3c**	CLL	WuHu.1	(COVID-, 2 doses)	(COVID-, 3 doses)	<0·0001
**Fig 3c**	CLL	WuHu.1	(COVID-, 2 doses)	(COVID-, 4 doses)	<0·0001

**Fig 3c**	CLL	WuHu.1	(COVID-, 3 doses)	(COVID-, 4 doses)	0·33
**Fig 3c**	CLL	WuHu.1	(COVID+, 2 doses)	(COVID+, 3 doses)	1·00
**Fig 3c**	CLL	WuHu.1	(COVID+, 2 doses)	(COVID+, 4 doses)	1·00
**Fig 3c**	CLL	WuHu.1	(COVID+, 3 doses)	(COVID+, 4 doses)	1·00
**Fig 3c**	CLL	WuHu.1	(COVID-, 2 doses)	(COVID+, 2 doses)	0·16
**Fig 3c**	CLL	WuHu.1	(COVID-, 3 doses)	(COVID+, 3 doses)	1·00
**Fig 3c**	CLL	WuHu.1	(COVID-, 4 doses)	(COVID+, 4 doses)	1·00
**Fig 3c**	HC	BA.1	(COVID-, 2 doses)	(COVID-, 3 doses)	1·00
**Fig 3c**	HC	BA.1	(COVID+, 2 doses)	(COVID+, 3 doses)	1·00
**Fig 3c**	HC	BA.1	(COVID-, 2 doses)	(COVID+, 2 doses)	1·00
**Fig 3c**	HC	BA.1	(COVID-, 3 doses)	(COVID+, 3 doses)	0·0023
**Fig 3c**	PID	BA.1	(COVID-, 2 doses)	(COVID-, 3 doses)	0·14
**Fig 3c**	PID	BA.1	(COVID-, 2 doses)	(COVID-, 4 doses)	1·00
**Fig 3c**	PID	BA.1	(COVID-, 3 doses)	(COVID-, 4 doses)	1·00
**Fig 3c**	PID	BA.1	(COVID+, 2 doses)	(COVID+, 3 doses)	1·00
**Fig 3c**	PID	BA.1	(COVID+, 2 doses)	(COVID+, 4 doses)	1·00
**Fig 3c**	PID	BA.1	(COVID+, 3 doses)	(COVID+, 4 doses)	1·00
**Fig 3c**	PID	BA.1	(COVID-, 2 doses)	(COVID+, 2 doses)	1·00
**Fig 3c**	PID	BA.1	(COVID-, 3 doses)	(COVID+, 3 doses)	0·15
**Fig 3c**	PID	BA.1	(COVID-, 4 doses)	(COVID+, 4 doses)	1·00
**Fig 3c**	HIV	BA.1	(COVID-, 2 doses)	(COVID-, 3 doses)	0·87
**Fig 3c**	HIV	BA.1	(COVID+, 2 doses)	(COVID+, 3 doses)	1·00
**Fig 3c**	HIV	BA.1	(COVID-, 2 doses)	(COVID+, 2 doses)	1·00
**Fig 3c**	HIV	BA.1	(COVID-, 3 doses)	(COVID+, 3 doses)	0·23
**Fig 3c**	HSCT	BA.1	(COVID-, 2 doses)	(COVID-, 3 doses)	1·00
**Fig 3c**	HSCT	BA.1	(COVID-, 2 doses)	(COVID-, 4 doses)	<0·0001
**Fig 3c**	HSCT	BA.1	(COVID-, 3 doses)	(COVID-, 4 doses)	0·0038
**Fig 3c**	HSCT	BA.1	(COVID+, 2 doses)	(COVID+, 3 doses)	1·00
**Fig 3c**	HSCT	BA.1	(COVID+, 2 doses)	(COVID+, 4 doses)	1·00
**Fig 3c**	HSCT	BA.1	(COVID+, 3 doses)	(COVID+, 4 doses)	1·00
**Fig 3c**	HSCT	BA.1	(COVID-, 2 doses)	(COVID+, 2 doses)	1·00
**Fig 3c**	HSCT	BA.1	(COVID-, 3 doses)	(COVID+, 3 doses)	0·0056
**Fig 3c**	HSCT	BA.1	(COVID-, 4 doses)	(COVID+, 4 doses)	1·00
**Fig 3c**	SOT	BA.1	(COVID-, 2 doses)	(COVID-, 3 doses)	0·17
**Fig 3c**	SOT	BA.1	(COVID-, 2 doses)	(COVID-, 4 doses)	1·00
**Fig 3c**	SOT	BA.1	(COVID-, 3 doses)	(COVID-, 4 doses)	1·00
**Fig 3c**	SOT	BA.1	(COVID+, 2 doses)	(COVID+, 3 doses)	1·00
**Fig 3c**	SOT	BA.1	(COVID+, 2 doses)	(COVID+, 4 doses)	1·00
**Fig 3c**	SOT	BA.1	(COVID+, 3 doses)	(COVID+, 4 doses)	1·00
**Fig 3c**	SOT	BA.1	(COVID-, 2 doses)	(COVID+, 2 doses)	0·13
**Fig 3c**	SOT	BA.1	(COVID-, 3 doses)	(COVID+, 3 doses)	1·00
**Fig 3c**	SOT	BA.1	(COVID-, 4 doses)	(COVID+, 4 doses)	1·00
**Fig 3c**	CLL	BA.1	(COVID-, 2 doses)	(COVID-, 3 doses)	1·00
**Fig 3c**	CLL	BA.1	(COVID-, 2 doses)	(COVID-, 4 doses)	1·00
**Fig 3c**	CLL	BA.1	(COVID-, 3 doses)	(COVID-, 4 doses)	1·00
**Fig 3c**	CLL	BA.1	(COVID+, 2 doses)	(COVID+, 3 doses)	1·00
**Fig 3c**	CLL	BA.1	(COVID+, 2 doses)	(COVID+, 4 doses)	0·56
**Fig 3c**	CLL	BA.1	(COVID+, 3 doses)	(COVID+, 4 doses)	0·16
**Fig 3c**	CLL	BA.1	(COVID-, 2 doses)	(COVID+, 2 doses)	1·00
**Fig 3c**	CLL	BA.1	(COVID-, 3 doses)	(COVID+, 3 doses)	1·00
**Fig 3c**	CLL	BA.1	(COVID-, 4 doses)	(COVID+, 4 doses)	0·76
**Fig 4a**	HC	Wu-Hu.1	('3m', COVID-)	('3m', COVID+)	1·00
**Fig 4a**	HC	Wu-Hu.1	('6m', COVID-)	('6m', COVID+)	1·00
**Fig 4a**	HC	Wu-Hu.1	('9m', COVID-)	('9m', COVID+)	1·00
**Fig 4a**	PID	Wu-Hu.1	('3m', COVID-)	('3m', COVID+)	1·00
**Fig 4a**	PID	Wu-Hu.1	('6m', COVID-)	('6m', COVID+)	1·00
**Fig 4a**	PID	Wu-Hu.1	('9m', COVID-)	('9m', COVID+)	1·00

**Fig 4a**	HIV	Wu-Hu.1	('3m', COVID-)	('3m', COVID+)	1·00
**Fig 4a**	HIV	Wu-Hu.1	('6m', COVID-)	('6m', COVID+)	1·00
**Fig 4a**	HIV	Wu-Hu.1	('9m', COVID-)	('9m', COVID+)	1·00
**Fig 4a**	HSCT	Wu-Hu.1	('3m', COVID-)	('3m', COVID+)	1·00
**Fig 4a**	HSCT	Wu-Hu.1	('6m', COVID-)	('6m', COVID+)	1·00
**Fig 4a**	HSCT	Wu-Hu.1	('9m', COVID-)	('9m', COVID+)	0·63
**Fig 4a**	SOT	Wu-Hu.1	('3m', COVID-)	('3m', COVID+)	0·82
**Fig 4a**	SOT	Wu-Hu.1	('6m', COVID-)	('6m', COVID+)	1·00
**Fig 4a**	SOT	Wu-Hu.1	('9m', COVID-)	('9m', COVID+)	0·64
**Fig 4a**	CLL	Wu-Hu.1	('3m', COVID-)	('3m', COVID+)	0·90
**Fig 4a**	CLL	Wu-Hu.1	('6m', COVID-)	('6m', COVID+)	0·40
**Fig 4a**	CLL	Wu-Hu.1	('9m', COVID-)	('9m', COVID+)	0·80
**Fig 4a**	HC	BA.1	('3m', COVID-)	('3m', COVID+)	1·00
**Fig 4a**	HC	BA.1	('6m', COVID-)	('6m', COVID+)	1·00
**Fig 4a**	HC	BA.1	('9m', COVID-)	('9m', COVID+)	1·00
**Fig 4a**	PID	BA.1	('3m', COVID-)	('3m', COVID+)	1·00
**Fig 4a**	PID	BA.1	('6m', COVID-)	('6m', COVID+)	1·00
**Fig 4a**	PID	BA.1	('9m', COVID-)	('9m', COVID+)	1·00
**Fig 4a**	HIV	BA.1	('3m', COVID-)	('3m', COVID+)	1·00
**Fig 4a**	HIV	BA.1	('6m', COVID-)	('6m', COVID+)	1·00
**Fig 4a**	HIV	BA.1	('9m', COVID-)	('9m', COVID+)	1·00
**Fig 4a**	HSCT	BA.1	('3m', COVID-)	('3m', COVID+)	1·00
**Fig 4a**	HSCT	BA.1	('6m', COVID-)	('6m', COVID+)	1·00
**Fig 4a**	HSCT	BA.1	('9m', COVID-)	('9m', COVID+)	0·43
**Fig 4a**	SOT	BA.1	('3m', COVID-)	('3m', COVID+)	1·00
**Fig 4a**	SOT	BA.1	('6m', COVID-)	('6m', COVID+)	0·77
**Fig 4a**	SOT	BA.1	('9m', COVID-)	('9m', COVID+)	0·58
**Fig 4a**	CLL	BA.1	('3m', COVID-)	('3m', COVID+)	0·99
**Fig 4a**	CLL	BA.1	('6m', COVID-)	('6m', COVID+)	0·44
**Fig 4a**	CLL	BA.1	('9m', COVID-)	('9m', COVID+)	0·42

**Supp Fig 2**	HC: >60 yrs	Wu-Hu.1	35d	3m	0·0049
**Supp Fig 2**	HC: >60 yrs	Wu-Hu.1	35d	6m	0·0097
**Supp Fig 2**	HC: >60 yrs	Wu-Hu.1	6m	9m	<0·0001
**Supp Fig 2**	HC: >60 yrs	Wu-Hu.1	6m	12m	0·0010
**Supp Fig 2**	HC: >60 yrs	Wu-Hu.1	9m	12m	1·00
**Supp Fig 2**	HC: 40-59 yrs	Wu-Hu.1	35d	3m	0·0049
**Supp Fig 2**	HC: 40-59 yrs	Wu-Hu.1	35d	6m	0·0002
**Supp Fig 2**	HC: 40-59 yrs	Wu-Hu.1	6m	9m	0·0004
**Supp Fig 2**	HC: 40-59 yrs	Wu-Hu.1	6m	12m	<0·0001
**Supp Fig 2**	HC: 40-59 yrs	Wu-Hu.1	9m	12m	1·00
**Supp Fig 2**	HC: 18-39 yrs	Wu-Hu.1	35d	3m	0·0049
**Supp Fig 2**	HC: 18-39 yrs	Wu-Hu.1	35d	6m	0·0006
**Supp Fig 2**	HC: 18-39 yrs	Wu-Hu.1	6m	9m	0·0049
**Supp Fig 2**	HC: 18-39 yrs	Wu-Hu.1	6m	12m	0·0024
**Supp Fig 2**	HC: 18-39 yrs	Wu-Hu.1	9m	12m	0·87
**Supp Fig 2**	PID: CVID	Wu-Hu.1	35d	3m	0·063
**Supp Fig 2**	PID: CVID	Wu-Hu.1	35d	6m	0·75
**Supp Fig 2**	PID: CVID	Wu-Hu.1	6m	9m	0·0003
**Supp Fig 2**	PID: CVID	Wu-Hu.1	6m	12m	<0·0001
**Supp Fig 2**	PID: CVID	Wu-Hu.1	9m	12m	0·0011
**Supp Fig 2**	PID: XLA	Wu-Hu.1	35d	3m	1·00
**Supp Fig 2**	PID: XLA	Wu-Hu.1	35d	6m	1·00
**Supp Fig 2**	PID: XLA	Wu-Hu.1	6m	9m	1·00
**Supp Fig 2**	PID: XLA	Wu-Hu.1	6m	12m	1·00
**Supp Fig 2**	PID: XLA	Wu-Hu.1	9m	12m	1·00
**Supp Fig 2**	PID: Monogenic disease	Wu-Hu.1	35d	3m	1·00
**Supp Fig 2**	PID: Monogenic disease	Wu-Hu.1	35d	6m	0·34

**Supp Fig 2**	PID: Monogenic disease	Wu-Hu.1	6m	9m	1·00
**Supp Fig 2**	PID: Monogenic disease	Wu-Hu.1	6m	12m	0·94
**Supp Fig 2**	PID: Monogenic disease	Wu-Hu.1	9m	12m	1·00
**Supp Fig 2**	PID: CD4-cytopenia	Wu-Hu.1	35d	3m	0·62
**Supp Fig 2**	PID: CD4-cytopenia	Wu-Hu.1	35d	6m	0·039
**Supp Fig 2**	PID: CD4-cytopenia	Wu-Hu.1	6m	9m	0·078
**Supp Fig 2**	PID: CD4-cytopenia	Wu-Hu.1	6m	12m	0·078
**Supp Fig 2**	PID: CD4-cytopenia	Wu-Hu.1	9m	12m	0·37
**Supp Fig 2**	PID: Other	Wu-Hu.1	35d	3m	1·00
**Supp Fig 2**	PID: Other	Wu-Hu.1	35d	6m	0·31
**Supp Fig 2**	PID: Other	Wu-Hu.1	6m	9m	0·16
**Supp Fig 2**	PID: Other	Wu-Hu.1	6m	12m	0·16
**Supp Fig 2**	PID: Other	Wu-Hu.1	9m	12m	1·00
**Supp Fig 2**	HIV: ≤CD4 300	Wu-Hu.1	35d	3m	0·54
**Supp Fig 2**	HIV: ≤CD4 300	Wu-Hu.1	35d	6m	0·30
**Supp Fig 2**	HIV: ≤CD4 300	Wu-Hu.1	6m	9m	0·25
**Supp Fig 2**	HIV: ≤CD4 300	Wu-Hu.1	6m	12m	0·059
**Supp Fig 2**	HIV: ≤CD4 300	Wu-Hu.1	9m	12m	1·00
**Supp Fig 2**	HIV: >CD4 300	Wu-Hu.1	35d	3m	<0·0001
**Supp Fig 2**	HIV: >CD4 300	Wu-Hu.1	35d	6m	<0·0001
**Supp Fig 2**	HIV: >CD4 300	Wu-Hu.1	6m	9m	<0·0001
**Supp Fig 2**	HIV: >CD4 300	Wu-Hu.1	6m	12m	<0·0001
**Supp Fig 2**	HIV: >CD4 300	Wu-Hu.1	9m	12m	0·27
**Supp Fig 2**	HSCT: Early	Wu-Hu.1	35d	3m	1·00
**Supp Fig 2**	HSCT: Early	Wu-Hu.1	35d	6m	1·00
**Supp Fig 2**	HSCT: Early	Wu-Hu.1	6m	9m	1·00
**Supp Fig 2**	HSCT: Early	Wu-Hu.1	6m	12m	1·00
**Supp Fig 2**	HSCT: Early	Wu-Hu.1	9m	12m	1·00
**Supp Fig 2**	HSCT: Intermediate	Wu-Hu.1	35d	3m	0·90
**Supp Fig 2**	HSCT: Intermediate	Wu-Hu.1	35d	6m	1·00
**Supp Fig 2**	HSCT: Intermediate	Wu-Hu.1	6m	9m	0·31
**Supp Fig 2**	HSCT: Intermediate	Wu-Hu.1	6m	12m	0·31
**Supp Fig 2**	HSCT: Intermediate	Wu-Hu.1	9m	12m	0·14
**Supp Fig 2**	HSCT: Late	Wu-Hu.1	35d	3m	<0·0001
**Supp Fig 2**	HSCT: Late	Wu-Hu.1	35d	6m	<0·0001
**Supp Fig 2**	HSCT: Late	Wu-Hu.1	6m	9m	<0·0001
**Supp Fig 2**	HSCT: Late	Wu-Hu.1	6m	12m	<0·0001
**Supp Fig 2**	HSCT: Late	Wu-Hu.1	9m	12m	0·0044
**Supp Fig 2**	SOT: ≤6mo w/ MMF	Wu-Hu.1	35d	3m	1·00
**Supp Fig 2**	SOT: ≤6mo w/ MMF	Wu-Hu.1	35d	6m	1·00
**Supp Fig 2**	SOT: ≤6mo w/ MMF	Wu-Hu.1	6m	9m	0·038
**Supp Fig 2**	SOT: ≤6mo w/ MMF	Wu-Hu.1	6m	12m	0·038
**Supp Fig 2**	SOT: ≤6mo w/ MMF	Wu-Hu.1	9m	12m	0·025
**Supp Fig 2**	SOT: >6mo w/ MMF	Wu-Hu.1	35d	3m	0·34
**Supp Fig 2**	SOT: >6mo w/ MMF	Wu-Hu.1	35d	6m	0·090
**Supp Fig 2**	SOT: >6mo w/ MMF	Wu-Hu.1	6m	9m	0·13
**Supp Fig 2**	SOT: >6mo w/ MMF	Wu-Hu.1	6m	12m	0·025
**Supp Fig 2**	SOT: >6mo w/ MMF	Wu-Hu.1	9m	12m	0·025
**Supp Fig 2**	SOT: >6mo w/o MMF	Wu-Hu.1	35d	3m	1·00
**Supp Fig 2**	SOT: >6mo w/o MMF	Wu-Hu.1	35d	6m	1·00
**Supp Fig 2**	SOT: >6mo w/o MMF	Wu-Hu.1	6m	9m	0·015
**Supp Fig 2**	SOT: >6mo w/o MMF	Wu-Hu.1	6m	12m	0·0098
**Supp Fig 2**	SOT: >6mo w/o MMF	Wu-Hu.1	9m	12m	0·0049
**Supp Fig 2**	CLL: Ibrutinib	Wu-Hu.1	35d	3m	1·00
**Supp Fig 2**	CLL: Ibrutinib	Wu-Hu.1	35d	6m	1·00
**Supp Fig 2**	CLL: Ibrutinib	Wu-Hu.1	6m	9m	0·090
**Supp Fig 2**	CLL: Ibrutinib	Wu-Hu.1	6m	12m	0·059
**Supp Fig 2**	CLL: Ibrutinib	Wu-Hu.1	9m	12m	0·059

**Supp Fig 2**	CLL: Off Ibrutinib	Wu-Hu.1	35d	3m	1·00
**Supp Fig 2**	CLL: Off Ibrutinib	Wu-Hu.1	35d	6m	1·00
**Supp Fig 2**	CLL: Off Ibrutinib	Wu-Hu.1	6m	9m	0·22
**Supp Fig 2**	CLL: Off Ibrutinib	Wu-Hu.1	6m	12m	0·22
**Supp Fig 2**	CLL: Off Ibrutinib	Wu-Hu.1	9m	12m	0·22
**Supp Fig 2**	CLL: Indolent	Wu-Hu.1	35d	3m	1·00
**Supp Fig 2**	CLL: Indolent	Wu-Hu.1	35d	6m	1·00
**Supp Fig 2**	CLL: Indolent	Wu-Hu.1	6m	9m	0·0008
**Supp Fig 2**	CLL: Indolent	Wu-Hu.1	6m	12m	0·0004
**Supp Fig 2**	CLL: Indolent	Wu-Hu.1	9m	12m	0·018
**Supp Fig 2**	CLL: BR/FCR	Wu-Hu.1	35d	3m	1·00
**Supp Fig 2**	CLL: BR/FCR	Wu-Hu.1	35d	6m	1·00
**Supp Fig 2**	CLL: BR/FCR	Wu-Hu.1	6m	9m	0·0061
**Supp Fig 2**	CLL: BR/FCR	Wu-Hu.1	6m	12m	0·0006
**Supp Fig 2**	CLL: BR/FCR	Wu-Hu.1	9m	12m	0·054
**Supp Fig 3a**	HC: >60 yrs	BA.1	(COVID-, 2 doses)	(COVID-, 3 doses)	<0·0001
**Supp Fig 3a**	HC: >60 yrs	BA.1	(COVID-, 3 doses)	(COVID+, 3 doses)	0·0025
**Supp Fig 3a**	HC: 40-59 yrs	BA.1	(COVID-, 2 doses)	(COVID-, 3 doses)	<0·0001
**Supp Fig 3a**	HC: 40-59 yrs	BA.1	(COVID-, 3 doses)	(COVID+, 3 doses)	0·11
**Supp Fig 3a**	HC: 18-39 yrs	BA.1	(COVID-, 2 doses)	(COVID-, 3 doses)	0·0001
**Supp Fig 3a**	HC: 18-39 yrs	BA.1	(COVID-, 3 doses)	(COVID+, 3 doses)	0·0049
**Supp Fig 3a**	PID: CVID	BA.1	(COVID-, 2 doses)	(COVID-, 3 doses)	0·20
**Supp Fig 3a**	PID: CVID	BA.1	(COVID-, 2 doses)	(COVID-, 4 doses)	1·00
**Supp Fig 3a**	PID: CVID	BA.1	(COVID-, 3 doses)	(COVID-, 4 doses)	1·00
**Supp Fig 3a**	PID: CVID	BA.1	(COVID-, 3 doses)	(COVID+, 3 doses)	0·63
**Supp Fig 3a**	PID: CVID	BA.1	(COVID-, 4 doses)	(COVID+, 4 doses)	0·40
**Supp Fig 3a**	PID: CVID	BA.1	(COVID+, 3 doses)	(COVID+, 4 doses)	1·00
**Supp Fig 3a**	PID: CD4-cytopenia	BA.1	(COVID-, 2 doses)	(COVID-, 3 doses)	0·0005
**Supp Fig 3a**	PID: CD4-cytopenia	BA.1	(COVID-, 2 doses)	(COVID-, 4 doses)	0·0030
**Supp Fig 3a**	PID: CD4-cytopenia	BA.1	(COVID-, 3 doses)	(COVID-, 4 doses)	0·14
**Supp Fig 3a**	HIV: ≤CD4 300	BA.1	(COVID-, 2 doses)	(COVID-, 3 doses)	0·088
**Supp Fig 3a**	HIV: >CD4 300	BA.1	(COVID-, 2 doses)	(COVID-, 3 doses)	<0·0001
**Supp Fig 3a**	HIV: >CD4 300	BA.1	(COVID-, 2 doses)	(COVID+, 2 doses)	0·044
**Supp Fig 3a**	HIV: >CD4 300	BA.1	(COVID-, 3 doses)	(COVID+, 3 doses)	0·047
**Supp Fig 3a**	HIV: >CD4 300	BA.1	(COVID+, 2 doses)	(COVID+, 3 doses)	1·00
**Supp Fig 3a**	HSCT: Intermediate	BA.1	(COVID-, 2 doses)	(COVID-, 3 doses)	0·056
**Supp Fig 3a**	HSCT: Intermediate	BA.1	(COVID-, 2 doses)	(COVID-, 4 doses)	0·0080
**Supp Fig 3a**	HSCT: Intermediate	BA.1	(COVID-, 3 doses)	(COVID-, 4 doses)	0·25
**Supp Fig 3a**	HSCT: Late	BA.1	(COVID-, 2 doses)	(COVID-, 3 doses)	<0·0001
**Supp Fig 3a**	HSCT: Late	BA.1	(COVID-, 2 doses)	(COVID-, 4 doses)	<0·0001
**Supp Fig 3a**	HSCT: Late	BA.1	(COVID-, 3 doses)	(COVID-, 4 doses)	0·0006
**Supp Fig 3a**	HSCT: Late	BA.1	(COVID-, 3 doses)	(COVID+, 3 doses)	0·0042
**Supp Fig 3a**	SOT: ≤6mo w/ MMF	BA.1	(COVID-, 2 doses)	(COVID-, 3 doses)	0·068
**Supp Fig 3a**	SOT: ≤6mo w/ MMF	BA.1	(COVID-, 2 doses)	(COVID-, 4 doses)	0·0001
**Supp Fig 3a**	SOT: ≤6mo w/ MMF	BA.1	(COVID-, 3 doses)	(COVID-, 4 doses)	0·24
**Supp Fig 3a**	SOT: ≤6mo w/ MMF	BA.1	(COVID-, 4 doses)	(COVID+, 4 doses)	0·080
**Supp Fig 3a**	SOT: >6mo w/ MMF	BA.1	(COVID-, 2 doses)	(COVID-, 3 doses)	0·050
**Supp Fig 3a**	SOT: >6mo w/ MMF	BA.1	(COVID-, 2 doses)	(COVID-, 4 doses)	0·0018
**Supp Fig 3a**	SOT: >6mo w/ MMF	BA.1	(COVID-, 3 doses)	(COVID-, 4 doses)	0·082
**Supp Fig 3a**	SOT: >6mo w/o MMF	BA.1	(COVID-, 2 doses)	(COVID-, 3 doses)	0·0001
**Supp Fig 3a**	SOT: >6mo w/o MMF	BA.1	(COVID-, 2 doses)	(COVID-, 4 doses)	0·0004
**Supp Fig 3a**	SOT: >6mo w/o MMF	BA.1	(COVID-, 3 doses)	(COVID-, 4 doses)	0·017
**Supp Fig 3a**	CLL: Ibrutinib	BA.1	(COVID-, 2 doses)	(COVID-, 3 doses)	0·16
**Supp Fig 3a**	CLL: Ibrutinib	BA.1	(COVID-, 2 doses)	(COVID-, 4 doses)	0·051
**Supp Fig 3a**	CLL: Ibrutinib	BA.1	(COVID-, 3 doses)	(COVID-, 4 doses)	0·86
**Supp Fig 3a**	CLL: Off Ibrutinib	BA.1	(COVID-, 2 doses)	(COVID-, 3 doses)	0·41
**Supp Fig 3a**	CLL: Indolent	BA.1	(COVID-, 2 doses)	(COVID-, 3 doses)	0·0002

**Supp Fig 3a**	CLL: Indolent	BA.1	(COVID-, 2 doses)	(COVID-, 4 doses)	<0·0001
**Supp Fig 3a**	CLL: Indolent	BA.1	(COVID-, 3 doses)	(COVID-, 4 doses)	0·26
**Supp Fig 3a**	CLL: Indolent	BA.1	(COVID-, 3 doses)	(COVID+, 3 doses)	0·62
**Supp Fig 3a**	CLL: BR/FCR	BA.1	(COVID-, 2 doses)	(COVID-, 3 doses)	0·010
**Supp Fig 3a**	CLL: BR/FCR	BA.1	(COVID-, 2 doses)	(COVID-, 4 doses)	0·0002
**Supp Fig 3a**	CLL: BR/FCR	BA.1	(COVID-, 3 doses)	(COVID-, 4 doses)	0·13
**Supp Fig 3b**	HC: >60 yrs	BA.1	(COVID-, 2 doses)	(COVID-, 3 doses)	<0·0001
**Supp Fig 3b**	HC: >60 yrs	BA.1	(COVID-, 3 doses)	(COVID+, 3 doses)	0·0050
**Supp Fig 3b**	HC: 40-59 yrs	BA.1	(COVID-, 2 doses)	(COVID-, 3 doses)	<0·0001
**Supp Fig 3b**	HC: 40-59 yrs	BA.1	(COVID-, 3 doses)	(COVID+, 3 doses)	0·26
**Supp Fig 3b**	HC: 18-39 yrs	BA.1	(COVID-, 2 doses)	(COVID-, 3 doses)	0·0001
**Supp Fig 3b**	HC: 18-39 yrs	BA.1	(COVID-, 3 doses)	(COVID+, 3 doses)	0·016
**Supp Fig 3b**	PID: CVID	BA.1	(COVID-, 2 doses)	(COVID-, 3 doses)	0·19
**Supp Fig 3b**	PID: CVID	BA.1	(COVID-, 2 doses)	(COVID-, 4 doses)	1·00
**Supp Fig 3b**	PID: CVID	BA.1	(COVID-, 3 doses)	(COVID-, 4 doses)	1·00
**Supp Fig 3b**	PID: CVID	BA.1	(COVID-, 3 doses)	(COVID+, 3 doses)	0·51
**Supp Fig 3b**	PID: CVID	BA.1	(COVID-, 4 doses)	(COVID+, 4 doses)	0·28
**Supp Fig 3b**	PID: CVID	BA.1	(COVID+, 3 doses)	(COVID+, 4 doses)	1·00
**Supp Fig 3b**	PID: CD4-cytopenia	BA.1	(COVID-, 2 doses)	(COVID-, 3 doses)	0·0005
**Supp Fig 3b**	PID: CD4-cytopenia	BA.1	(COVID-, 2 doses)	(COVID-, 4 doses)	0·0030
**Supp Fig 3b**	PID: CD4-cytopenia	BA.1	(COVID-, 3 doses)	(COVID-, 4 doses)	0·091
**Supp Fig 3b**	HIV: ≤CD4 300	BA.1	(COVID-, 2 doses)	(COVID-, 3 doses)	0·043
**Supp Fig 3b**	HIV: >CD4 300	BA.1	(COVID-, 2 doses)	(COVID-, 3 doses)	<0·0001
**Supp Fig 3b**	HIV: >CD4 300	BA.1	(COVID-, 2 doses)	(COVID+, 2 doses)	0·023
**Supp Fig 3b**	HIV: >CD4 300	BA.1	(COVID-, 3 doses)	(COVID+, 3 doses)	0·023
**Supp Fig 3b**	HIV: >CD4 300	BA.1	(COVID+, 2 doses)	(COVID+, 3 doses)	1·00
**Supp Fig 3b**	HSCT: Intermediate	BA.1	(COVID-, 2 doses)	(COVID-, 3 doses)	0·0047
**Supp Fig 3b**	HSCT: Intermediate	BA.1	(COVID-, 2 doses)	(COVID-, 4 doses)	0·014
**Supp Fig 3b**	HSCT: Intermediate	BA.1	(COVID-, 3 doses)	(COVID-, 4 doses)	0·38
**Supp Fig 3b**	HSCT: Late	BA.1	(COVID-, 2 doses)	(COVID-, 3 doses)	<0·0001
**Supp Fig 3b**	HSCT: Late	BA.1	(COVID-, 2 doses)	(COVID-, 4 doses)	<0·0001
**Supp Fig 3b**	HSCT: Late	BA.1	(COVID-, 3 doses)	(COVID-, 4 doses)	0·0003
**Supp Fig 3b**	HSCT: Late	BA.1	(COVID-, 3 doses)	(COVID+, 3 doses)	0·0032
**Supp Fig 3b**	SOT: ≤6mo w/ MMF	BA.1	(COVID-, 2 doses)	(COVID-, 3 doses)	0·080
**Supp Fig 3b**	SOT: ≤6mo w/ MMF	BA.1	(COVID-, 2 doses)	(COVID-, 4 doses)	0·0003
**Supp Fig 3b**	SOT: ≤6mo w/ MMF	BA.1	(COVID-, 3 doses)	(COVID-, 4 doses)	0·56
**Supp Fig 3b**	SOT: ≤6mo w/ MMF	BA.1	(COVID-, 4 doses)	(COVID+, 4 doses)	0·17
**Supp Fig 3b**	SOT: >6mo w/ MMF	BA.1	(COVID-, 2 doses)	(COVID-, 3 doses)	0·0019
**Supp Fig 3b**	SOT: >6mo w/ MMF	BA.1	(COVID-, 2 doses)	(COVID-, 4 doses)	0·0012
**Supp Fig 3b**	SOT: >6mo w/ MMF	BA.1	(COVID-, 3 doses)	(COVID-, 4 doses)	0·069
**Supp Fig 3b**	SOT: >6mo w/o MMF	BA.1	(COVID-, 2 doses)	(COVID-, 3 doses)	<0·0001
**Supp Fig 3b**	SOT: >6mo w/o MMF	BA.1	(COVID-, 2 doses)	(COVID-, 4 doses)	0·0003
**Supp Fig 3b**	SOT: >6mo w/o MMF	BA.1	(COVID-, 3 doses)	(COVID-, 4 doses)	0·022
**Supp Fig 3b**	CLL: Ibrutinib	BA.1	(COVID-, 2 doses)	(COVID-, 3 doses)	0·074
**Supp Fig 3b**	CLL: Ibrutinib	BA.1	(COVID-, 2 doses)	(COVID-, 4 doses)	0·021
**Supp Fig 3b**	CLL: Ibrutinib	BA.1	(COVID-, 3 doses)	(COVID-, 4 doses)	1·00
**Supp Fig 3b**	CLL: Off Ibrutinib	BA.1	(COVID-, 2 doses)	(COVID-, 3 doses)	0·18
**Supp Fig 3b**	CLL: Indolent	BA.1	(COVID-, 2 doses)	(COVID-, 3 doses)	0·0001
**Supp Fig 3b**	CLL: Indolent	BA.1	(COVID-, 2 doses)	(COVID-, 4 doses)	<0·0001
**Supp Fig 3b**	CLL: Indolent	BA.1	(COVID-, 3 doses)	(COVID-, 4 doses)	0·32
**Supp Fig 3b**	CLL: Indolent	BA.1	(COVID-, 3 doses)	(COVID+, 3 doses)	0·55
**Supp Fig 3b**	CLL: BR/FCR	BA.1	(COVID-, 2 doses)	(COVID-, 3 doses)	0·0044
**Supp Fig 3b**	CLL: BR/FCR	BA.1	(COVID-, 2 doses)	(COVID-, 4 doses)	0·0002
**Supp Fig 3b**	CLL: BR/FCR	BA.1	(COVID-, 3 doses)	(COVID-, 4 doses)	0·18
**Supp Fig 4a**	HC: >60 yrs	Wu-Hu.1	(COVID-, 2 doses)	(COVID-, 3 doses)	<0·0001
**Supp Fig 4a**	HC: >60 yrs	Wu-Hu.1	(COVID-, 3 doses)	(COVID+, 3 doses)	0·012
**Supp Fig 4a**	HC: 40-59 yrs	Wu-Hu.1	(COVID-, 2 doses)	(COVID-, 3 doses)	<0·0001
**Supp Fig 4a**	HC: 40-59 yrs	Wu-Hu.1	(COVID-, 3 doses)	(COVID+, 3 doses)	0·16

**Supp Fig 4a**	HC: 18-39 yrs	Wu-Hu.1	(COVID-, 2 doses)	(COVID-, 3 doses)	0·0002
**Supp Fig 4a**	HC: 18-39 yrs	Wu-Hu.1	(COVID-, 3 doses)	(COVID+, 3 doses)	0·041
**Supp Fig 4a**	PID: CVID	Wu-Hu.1	(COVID-, 2 doses)	(COVID-, 3 doses)	0·0091
**Supp Fig 4a**	PID: CVID	Wu-Hu.1	(COVID-, 2 doses)	(COVID-, 4 doses)	0·61
**Supp Fig 4a**	PID: CVID	Wu-Hu.1	(COVID-, 3 doses)	(COVID-, 4 doses)	1·00
**Supp Fig 4a**	PID: CVID	Wu-Hu.1	(COVID-, 3 doses)	(COVID+, 3 doses)	1·00
**Supp Fig 4a**	PID: CVID	Wu-Hu.1	(COVID-, 4 doses)	(COVID+, 4 doses)	1·00
**Supp Fig 4a**	PID: CVID	Wu-Hu.1	(COVID+, 3 doses)	(COVID+, 4 doses)	1·00
**Supp Fig 4a**	PID: CD4-cytopenia	Wu-Hu.1	(COVID-, 2 doses)	(COVID-, 3 doses)	0·0005
**Supp Fig 4a**	PID: CD4-cytopenia	Wu-Hu.1	(COVID-, 2 doses)	(COVID-, 4 doses)	0·0030
**Supp Fig 4a**	PID: CD4-cytopenia	Wu-Hu.1	(COVID-, 3 doses)	(COVID-, 4 doses)	0·61
**Supp Fig 4a**	HIV: ≤CD4 300	Wu-Hu.1	(COVID-, 2 doses)	(COVID-, 3 doses)	0·055
**Supp Fig 4a**	HIV: >CD4 300	Wu-Hu.1	(COVID-, 2 doses)	(COVID-, 3 doses)	<0·0001
**Supp Fig 4a**	HIV: >CD4 300	Wu-Hu.1	(COVID-, 2 doses)	(COVID+, 2 doses)	0·023
**Supp Fig 4a**	HIV: >CD4 300	Wu-Hu.1	(COVID-, 3 doses)	(COVID+, 3 doses)	0·036
**Supp Fig 4a**	HIV: >CD4 300	Wu-Hu.1	(COVID+, 2 doses)	(COVID+, 3 doses)	1·00
**Supp Fig 4a**	HSCT: Intermediate	Wu-Hu.1	(COVID-, 2 doses)	(COVID-, 3 doses)	0·056
**Supp Fig 4a**	HSCT: Intermediate	Wu-Hu.1	(COVID-, 2 doses)	(COVID-, 4 doses)	0·088
**Supp Fig 4a**	HSCT: Intermediate	Wu-Hu.1	(COVID-, 3 doses)	(COVID-, 4 doses)	0·99
**Supp Fig 4a**	HSCT: Late	Wu-Hu.1	(COVID-, 2 doses)	(COVID-, 3 doses)	<0·0001
**Supp Fig 4a**	HSCT: Late	Wu-Hu.1	(COVID-, 2 doses)	(COVID-, 4 doses)	<0·0001
**Supp Fig 4a**	HSCT: Late	Wu-Hu.1	(COVID-, 3 doses)	(COVID-, 4 doses)	0·0012
**Supp Fig 4a**	HSCT: Late	Wu-Hu.1	(COVID-, 3 doses)	(COVID+, 3 doses)	0·049
**Supp Fig 4a**	SOT: ≤6mo w/ MMF	Wu-Hu.1	(COVID-, 2 doses)	(COVID-, 3 doses)	0·15
**Supp Fig 4a**	SOT: ≤6mo w/ MMF	Wu-Hu.1	(COVID-, 2 doses)	(COVID-, 4 doses)	0·18
**Supp Fig 4a**	SOT: ≤6mo w/ MMF	Wu-Hu.1	(COVID-, 3 doses)	(COVID-, 4 doses)	1·00
**Supp Fig 4a**	SOT: ≤6mo w/ MMF	Wu-Hu.1	(COVID-, 4 doses)	(COVID+, 4 doses)	0·33
**Supp Fig 4a**	SOT: >6mo w/ MMF	Wu-Hu.1	(COVID-, 2 doses)	(COVID-, 3 doses)	0·011
**Supp Fig 4a**	SOT: >6mo w/ MMF	Wu-Hu.1	(COVID-, 2 doses)	(COVID-, 4 doses)	0·14
**Supp Fig 4a**	SOT: >6mo w/ MMF	Wu-Hu.1	(COVID-, 3 doses)	(COVID-, 4 doses)	1·00
**Supp Fig 4a**	SOT: >6mo w/o MMF	Wu-Hu.1	(COVID-, 2 doses)	(COVID-, 3 doses)	0·0029
**Supp Fig 4a**	SOT: >6mo w/o MMF	Wu-Hu.1	(COVID-, 2 doses)	(COVID-, 4 doses)	0·0012
**Supp Fig 4a**	SOT: >6mo w/o MMF	Wu-Hu.1	(COVID-, 3 doses)	(COVID-, 4 doses)	0·017
**Supp Fig 4a**	CLL: Ibrutinib	Wu-Hu.1	(COVID-, 2 doses)	(COVID-, 3 doses)	1·00
**Supp Fig 4a**	CLL: Ibrutinib	Wu-Hu.1	(COVID-, 2 doses)	(COVID-, 4 doses)	1·00
**Supp Fig 4a**	CLL: Ibrutinib	Wu-Hu.1	(COVID-, 3 doses)	(COVID-, 4 doses)	1·00
**Supp Fig 4a**	CLL: Off Ibrutinib	Wu-Hu.1	(COVID-, 2 doses)	(COVID-, 3 doses)	0·66
**Supp Fig 4a**	CLL: Indolent	Wu-Hu.1	(COVID-, 2 doses)	(COVID-, 3 doses)	<0·0001
**Supp Fig 4a**	CLL: Indolent	Wu-Hu.1	(COVID-, 2 doses)	(COVID-, 4 doses)	<0·0001
**Supp Fig 4a**	CLL: Indolent	Wu-Hu.1	(COVID-, 3 doses)	(COVID-, 4 doses)	0·16
**Supp Fig 4a**	CLL: Indolent	Wu-Hu.1	(COVID-, 3 doses)	(COVID+, 3 doses)	1·00
**Supp Fig 4a**	CLL: BR/FCR	Wu-Hu.1	(COVID-, 2 doses)	(COVID-, 3 doses)	0·015
**Supp Fig 4a**	CLL: BR/FCR	Wu-Hu.1	(COVID-, 2 doses)	(COVID-, 4 doses)	0·0002
**Supp Fig 4a**	CLL: BR/FCR	Wu-Hu.1	(COVID-, 3 doses)	(COVID-, 4 doses)	0·18
**Supp Fig 4b**	HC: >60 yrs	BA.1	(COVID-, 2 doses)	(COVID-, 3 doses)	0·39
**Supp Fig 4b**	HC: >60 yrs	BA.1	(COVID-, 3 doses)	(COVID+, 3 doses)	0·90
**Supp Fig 4b**	HC: 40-59 yrs	BA.1	(COVID-, 2 doses)	(COVID-, 3 doses)	0·37
**Supp Fig 4b**	HC: 40-59 yrs	BA.1	(COVID-, 3 doses)	(COVID+, 3 doses)	0·045
**Supp Fig 4b**	HC: 18-39 yrs	BA.1	(COVID-, 2 doses)	(COVID-, 3 doses)	1·00
**Supp Fig 4b**	HC: 18-39 yrs	BA.1	(COVID-, 3 doses)	(COVID+, 3 doses)	0·097
**Supp Fig 4b**	PID: CVID	BA.1	(COVID-, 2 doses)	(COVID-, 3 doses)	0·15
**Supp Fig 4b**	PID: CVID	BA.1	(COVID-, 2 doses)	(COVID-, 4 doses)	1·00
**Supp Fig 4b**	PID: CVID	BA.1	(COVID-, 3 doses)	(COVID-, 4 doses)	1·00
**Supp Fig 4b**	PID: CVID	BA.1	(COVID-, 3 doses)	(COVID+, 3 doses)	1·00
**Supp Fig 4b**	PID: CVID	BA.1	(COVID-, 4 doses)	(COVID+, 4 doses)	1·00
**Supp Fig 4b**	PID: CVID	BA.1	(COVID+, 3 doses)	(COVID+, 4 doses)	1·00
**Supp Fig 4b**	PID: CD4-cytopenia	BA.1	(COVID-, 2 doses)	(COVID-, 3 doses)	0·49
**Supp Fig 4b**	PID: CD4-cytopenia	BA.1	(COVID-, 2 doses)	(COVID-, 4 doses)	0·40

**Supp Fig 4b**	PID: CD4-cytopenia	BA.1	(COVID-, 3 doses)	(COVID-, 4 doses)	1·00
**Supp Fig 4b**	HIV: ≤CD4 300	BA.1	(COVID-, 2 doses)	(COVID-, 3 doses)	0·81
**Supp Fig 4b**	HIV: >CD4 300	BA.1	(COVID-, 2 doses)	(COVID-, 3 doses)	1·00
**Supp Fig 4b**	HIV: >CD4 300	BA.1	(COVID-, 2 doses)	(COVID+, 2 doses)	1·00
**Supp Fig 4b**	HIV: >CD4 300	BA.1	(COVID-, 3 doses)	(COVID+, 3 doses)	0·39
**Supp Fig 4b**	HIV: >CD4 300	BA.1	(COVID+, 2 doses)	(COVID+, 3 doses)	1·00
**Supp Fig 4b**	HSCT: Intermediate	BA.1	(COVID-, 2 doses)	(COVID-, 3 doses)	1·00
**Supp Fig 4b**	HSCT: Intermediate	BA.1	(COVID-, 2 doses)	(COVID-, 4 doses)	0·011
**Supp Fig 4b**	HSCT: Intermediate	BA.1	(COVID-, 3 doses)	(COVID-, 4 doses)	0·35
**Supp Fig 4b**	HSCT: Late	BA.1	(COVID-, 2 doses)	(COVID-, 3 doses)	1·00
**Supp Fig 4b**	HSCT: Late	BA.1	(COVID-, 2 doses)	(COVID-, 4 doses)	0·0009
**Supp Fig 4b**	HSCT: Late	BA.1	(COVID-, 3 doses)	(COVID-, 4 doses)	0·016
**Supp Fig 4b**	HSCT: Late	BA.1	(COVID-, 3 doses)	(COVID+, 3 doses)	0·017
**Supp Fig 4b**	SOT: ≤6mo w/ MMF	BA.1	(COVID-, 2 doses)	(COVID-, 3 doses)	1·00
**Supp Fig 4b**	SOT: ≤6mo w/ MMF	BA.1	(COVID-, 2 doses)	(COVID-, 4 doses)	1·00
**Supp Fig 4b**	SOT: ≤6mo w/ MMF	BA.1	(COVID-, 3 doses)	(COVID-, 4 doses)	1·00
**Supp Fig 4b**	SOT: ≤6mo w/ MMF	BA.1	(COVID-, 4 doses)	(COVID+, 4 doses)	1·00
**Supp Fig 4b**	SOT: >6mo w/ MMF	BA.1	(COVID-, 2 doses)	(COVID-, 3 doses)	0·020
**Supp Fig 4b**	SOT: >6mo w/ MMF	BA.1	(COVID-, 2 doses)	(COVID-, 4 doses)	0·30
**Supp Fig 4b**	SOT: >6mo w/ MMF	BA.1	(COVID-, 3 doses)	(COVID-, 4 doses)	1·00
**Supp Fig 4b**	SOT: >6mo w/o MMF	BA.1	(COVID-, 2 doses)	(COVID-, 3 doses)	1·00
**Supp Fig 4b**	SOT: >6mo w/o MMF	BA.1	(COVID-, 2 doses)	(COVID-, 4 doses)	0·31
**Supp Fig 4b**	SOT: >6mo w/o MMF	BA.1	(COVID-, 3 doses)	(COVID-, 4 doses)	0·10
**Supp Fig 4b**	CLL: Ibrutinib	BA.1	(COVID-, 2 doses)	(COVID-, 3 doses)	0·25
**Supp Fig 4b**	CLL: Ibrutinib	BA.1	(COVID-, 2 doses)	(COVID-, 4 doses)	0·051
**Supp Fig 4b**	CLL: Ibrutinib	BA.1	(COVID-, 3 doses)	(COVID-, 4 doses)	0·86
**Supp Fig 4b**	CLL: Off Ibrutinib	BA.1	(COVID-, 2 doses)	(COVID-, 3 doses)	0·23
**Supp Fig 4b**	CLL: Indolent	BA.1	(COVID-, 2 doses)	(COVID-, 3 doses)	0·86
**Supp Fig 4b**	CLL: Indolent	BA.1	(COVID-, 2 doses)	(COVID-, 4 doses)	0·23
**Supp Fig 4b**	CLL: Indolent	BA.1	(COVID-, 3 doses)	(COVID-, 4 doses)	0·39
**Supp Fig 4b**	CLL: Indolent	BA.1	(COVID-, 3 doses)	(COVID+, 3 doses)	1·00
**Supp Fig 4b**	CLL: BR/FCR	BA.1	(COVID-, 2 doses)	(COVID-, 3 doses)	1·00
**Supp Fig 4b**	CLL: BR/FCR	BA.1	(COVID-, 2 doses)	(COVID-, 4 doses)	0·50
**Supp Fig 4b**	CLL: BR/FCR	BA.1	(COVID-, 3 doses)	(COVID-, 4 doses)	1·00
**Supp Fig 5**	HC	Nucleocapsid	COVID+	COVID-	<0·0001
**Supp Fig 5**	PID	Nucleocapsid	COVID+	COVID-	<0·0001
**Supp Fig 5**	HIV	Nucleocapsid	COVID+	COVID-	0·0075
**Supp Fig 5**	HSCT	Nucleocapsid	COVID+	COVID-	<0·0001
**Supp Fig 5**	SOT	Nucleocapsid	COVID+	COVID-	0·0002
**Supp Fig 5**	CLL	Nucleocapsid	COVID+	COVID-	0·0064

Supplementary Table S1. Non-dichotomized p-values

	Drop-outs		Reconsented study cohort		Statistics
**Study subjects [n, %]**	Males	Females	Males	Females	**p-value**
HC	14 (43·8%)	18 (56·2%)	25 (43·1%)	33 (56·9%)	0·0489
PID	13 (48·1%)	14 (51·9%)	22 (34·9%)	41 (65·1%)	0·0004
HIV	23 (65·7%)	12 (34·3%)	32 (58·2%)	23 (41·8%)	0·0895
HSCT	15 (53·6%)	13 (46·4%)	32 (51·6%)	30 (48·4%)	0·0137
SOT	19 (52·8%)	17 (47·2%)	27 (50·9%)	26 (49·1%)	0·2221
CLL	19 (76·0%)	6 (24·0%)	41 (63·1%)	24 (36·9%)	0·0014
**Age in years [median, IQR]**					**p-value**
HC	35·5 (28·0-51·0)	55·0 (36·0-69·0)	55·0 (36·0-69·0)	57·0 (43·0-68·0)	0·0759
PID	38·0 (31·0-42·0)	47·0 (38·3-53·0)	47·0 (38·3-53·0)	55·0 (47·0-66·0)	0·0002
HIV	53·0 (40·5-60·5)	58·5 (49·0-68·5)	58·5 (49·0-68·5)	49·0 (43·0-62·5)	0·0563
HSCT	50·0 (39·0-57·5)	62·0 (54·8-68·0)	62·0 (54·8-68·0)	60·5 (50·3-67·8)	0·0006
SOT	55·0 (48·5-65·0)	59·0 (51·5-64·5)	59·0 (51·5-64·5)	53·5 (43·0-64·0)	0·7570
CLL	63·0 (59·0-74·5)	70·0 (63·0-74·0)	70·0 (63·0-74·0)	75·0 (69·8-79·5)	0·0473
**Ab titres at day 35 in AU/ml [geometric mean, geometric SD]**					**GMR (95% CI), p-value**
HC	3452·5 (2·4)	2800·4 (1·9)	2215·8 (2·6)	1972·9 (2·1)	0·68 (0·48, 0·95), 0·030
PID	35·6 (91·1)	78·0 (62·1)	23·8 (45·7)	102·8 (27·0)	1·21 (0·18, 8·23), 0·835
HIV	1565·0 (2·4)	1450·0 (2·8)	1790·4 (6·1)	1236·2 (8·0)	1·02 (0·56, 1·86), 0·965
HSCT	463·6 (47·6)	1131·1 (41·7)	243·6 (33·3)	476·4 (34·9)	0·46 (0·09, 2·50), 0·361
SOT	13·2 (41·5)	4·9 (22·2)	2·6 (22·2)	4·6 (26·5)	0·41 (0·10, 1·72), 0·220
CLL	6·5 (27·6)	0·4 (1·0)	14·7 (19·6)	30·5 (19·4)	4·81 (0·94, 24·68), 0·056

For sex, a two-sided binomial test with the null hypothesis that the proportion of females is the same for the reconsented study and those who dropped out was used. For age and antibody titres, p-values were calculated using Mann-Whitney U test and Independent t-test, respectively. For all statistics, study-group comparisons were made (male + female) between drop-outs and reconsented study group. GMR = geometric mean ratio, CI = Confidence interval, IQR = interquartile range

Supplementary Table S2. Demographics and antibody titers in reconsented study subjects and drop-outs.

	Third dose		Fourth dose	
	BNT162b2 (Comirnaty, Pfizer/BioNTech)	mRNA-1273 (Spikevax, Moderna)	BNT162b2 (Comirnaty, Pfizer/BioNTech)	mRNA-1273 (Spikevax, Moderna)
**HC subgroups**				
18-39 yrs	11	3		
40-59 yrs	11	8		
>60 yrs	20	1		
**PID subgroups**				
CVID	32	3	4	18
XLA	2	..	..	1
Monogenic disease	2	2	..	1
CD4-cytopenia	10	..	3	3
Other	5	..	..	..
**HIV subgroups**				
≤CD4 300	7	4		
>CD4 300	26	8		
**HSCT subgroups**				
Early	4	..	1	3
Intermediate	10	..	4	4
Late	41	1	3	24
**SOT subgroups**				
≤6mo	30	..	3	12
>6mo w/ MMF	19	..	4	8
>6mo w/o MMF	34	..	6	11
**CLL subgroups**				
Ibrutinib	15	..	1	12
Off Ibrutinib	6	2	1	4
Indolent	23	2	4	12
BR/FCR	14	..	1	13

Supplementary Table S3. Number of administered 3rd and 4th vaccine doses
